# Tailoring Tumor Cell Golgi Apparatus‐Targeting Self‐Assembled Peptide for Effective Immunotherapy via Reshaping MIF‐Mediated Immunosuppressive Network

**DOI:** 10.1002/advs.202415133

**Published:** 2025-02-05

**Authors:** Xiang Li, Chengxinqiao Wang, Junhan Liu, Guifang Deng, Yongqiang Deng, Fang Hu, Yupeng Wang, Dongfang Zhou

**Affiliations:** ^1^ NMPA Key Laboratory for Research and Evaluation of Drug Metabolism & Guangdong Provincial Key Laboratory of New Drug Screening & Guangdong‐Hongkong‐Macao Joint Laboratory for New Drug Screening School of Pharmaceutical Sciences Southern Medical University Guangzhou 510 515 P. R. China; ^2^ Department of Ultrasonic Diagnosis & Orthopedic and Traumatology Zhujiang Hospital Southern Medical University Guangzhou 510 515 P. R. China; ^3^ Department of Pathophysiology Guangdong Provincial Key Laboratory of Proteomics School of Basic Medical Sciences Southern Medical University Guangzhou 510 515 P. R. China; ^4^ Biomaterials Research Center School of Biomedical Engineering Southern Medical University Guangzhou 510 515 P. R. China; ^5^ Key Laboratory of Mental Health of the Ministry of Education Southern Medical University Guangzhou 510 515 P. R. China

**Keywords:** Golgi apparatus, immunotherapy, migration inhibitory factor, self‐assembled peptide, tumor immunosuppressive network

## Abstract

The immunosuppressive network formed by the enhanced crosstalk between tumor cells and various types of immune cells may ultimately lead to the formation of tumor immunosuppressive microenvironment (TIME). The Golgi apparatus (GA) of tumor cells is a key organelle in the formation of a tumor immunosuppressive network. However, there are no studies to show whether interfering with the GA of tumor cells can reshape the immunosuppressive network to enhance the effectiveness of immunotherapy. Therefore, the tumor cell GA‐targeting self‐assembled peptide (NF‐1) is tailored, and confirmed that NF‐1 treatment can achieve an effective immunotherapy and found that tumor cell‐derived GA‐dependent migration inhibitory factor (MIF) mediates the formation of immunosuppressive network in breast cancer (BRCA) through multi‐omics analysis, in vivo, and in vitro experiments. NF‐1 treatment‐induced MIF reduction can reshape the immunosuppressive network and convert a “cold” tumor into a “hot” tumor, thus enabling immunotherapy in BRCA and enhancing the ICB efficacy in colon adenocarcinoma (COAD). This study presents a general strategy for interfering with tumor GA for effective immunotherapy in BRCA, COAD, and other cancers characterized by a “cold” immune microenvironment.

## Graphical abstract

A self‐assembled peptide (NF‐1) targeting tumor cell Golgi apparatus (GA) is tailored for effective immunotherapy via reshaping the MIF‐mediated immunosuppressive network. After targeting tumor cell GA, the morphology of NF‐1 is changed from micelles to nano‐fibers under the action of furin, thus inducing cell apoptosis as well as GA dysfunction. Especially, the protein P115 on the GA is blocked, which in turn blocks the transporter of migration inhibitory factor (MIF), further reshaping the MIF‐mediated immunosuppressive network between tumor cells and various immune cells in the tumor microenvironment.

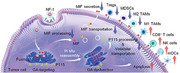

## 1 Introduction

Immunotherapy, primarily through immune checkpoint blockade (ICB) to inhibit immunosuppressive signaling, has become the cornerstone of cancer therapy.^[^ ^1^ ^]^ However, ICB therapy has shown limited effectiveness for “cold” tumors with a tumor immunosuppressive microenvironment (TIME).^[^ ^2^ ^]^ According to Li and Stanger's hypothesis,^[^ ^3^ ^]^ tumor cells occupy the top position in a signaling hierarchy within the TIME, indicating that the crosstalk between tumor cells and immune cells plays a crucial role in TIME formation. Tumor cells may regulate a variety of immune cells through paracrine, autocrine, and exocrine signals to form an immunosuppressive network that ultimately results in the TIME.^[^ ^4^ ^]^ However, most existing immunotherapy strategies target specific immune cells, immune checkpoints, or immune‐related pathways and often fail to address the need to reshape the immunosuppressive network, resulting in unsatisfactory immunotherapy outcomes. Therefore, it is necessary to develop therapeutic strategies that can reshape the immunosuppressive network by affecting crosstalk between tumor cells and multiple immune cells to enhance the effect of immunotherapy.

Numerous studies have demonstrated that the occurrence and progression of tumors and the malignant behavior of tumor cells are associated with the abnormal structure and function of subcellular organelles such as mitochondria, endoplasmic reticulum, lysosomes, etc.^[^ ^5^ ^]^ The Golgi apparatus (GA) serves as a central hub for protein and lipids modification and transportation.^[^ ^6^ ^]^ There is increasing evidence that tumor cells exploit the GA, resulting in defects in tumor cell proliferation and invasion, crosstalk between tumor cells and other cells, and immune regulation disorders.^[^ ^7^ ^]^ More importantly, tumor cells exhibit hyperactive secretory states and the GA is a major contributor to the tumor cell secretome as well as the crosstalk between tumor cells and immune cells, and aberrant GA dynamics can reshape the extracellular matrix and tumor microenvironment.^[^ ^8^ ^]^ A study has reported that treating T cells with exogenous H_2_S would overcome Golgi stress and restore GA function to enhance antitumor T cell response, highlighting the Golgi network as a previously unidentified therapeutic target for enhancing the efficacy of cancer immunotherapy.^[^ ^9^ ^]^ It has been reported that the GA may be a potential target of immunosuppression for producing several immunosuppressive cytokines, and there have been studies to develop biomaterials targeting the GA for co‐delivery of immunomodulators to achieve immunotherapy, highlighting its immunotherapy potential in melanoma.^[^ ^10^ ^]^ Thus, the GA of tumor cells may be a key organelle in the formation of an immunosuppressive network of tumors. However, there is still no research to show whether interfering with the GA of tumor cells can regulate the immunosuppressive network by influencing the crosstalk between tumor cells and multiple immune cells, and ultimately enhance the effect of immunotherapy.

In light of the unresolved challenges and the pressing need for an effective therapeutic strategy targeting the tumor immunosuppressive network, we have developed an intervention approach by tailoring a tumor cell‐derived GA‐targeting self‐assembled peptide (NF‐1). First, NF‐1 treatment can selectively and effectively disrupt tumor cell GA, resulting in a significant enhancement of immunotherapeutic efficacy in vitro and in vivo experiments. Second, through comprehensive multi‐omics analysis and in vitro experiments, tumor cell‐derived GA‐dependent migration inhibitory factor (MIF) was identified to play a crucial role in mediating the formation of an immunosuppressive network in breast cancer (BRCA). As a result, NF‐1 treatment can convert “cold” tumors into “hot” tumors by reshaping the MIF‐mediated immunosuppressive network, NF‐1 treatment thus enabling effective immunotherapy in BRCA and enhancing the ICB efficacy in colon adenocarcinoma (COAD).

## 2 Results and Discussion

### 2.1 Tumor Cell GA‐Targeting Self‐Assembled Peptide Induces GA Dysfunction and Cell Apoptosis

**Figure 1:**
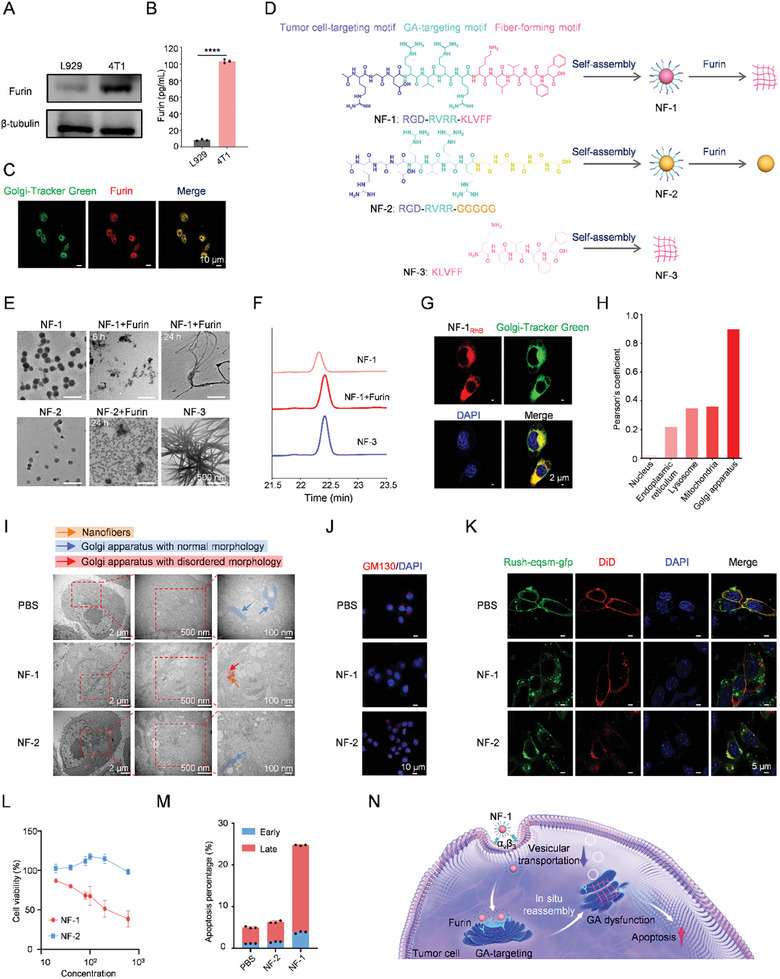
Tumor cell GA‐targeting self‐assembled peptide induces GA dysfunction and cell apoptosis. A) Furin expression in L929 and 4T1 cells. B) Quantitative analysis of furin in L929 and 4T1 cells by ELISA. C) CLSM images of co‐localization of furin with GA in 4T1 cells. D) Schematic diagram of the chemical structure and assembly of the functional targeting peptide NF‐1 and its two control peptides (NF‐2 and NF‐3) in aqueous solution. Different functional units of amino acid sequences were shown in various colors. Blue represents the tumor cell‐targeting motif; laurel green represents the GA‐targeting and furin‐responsive cleavage motif; pink represents the motif of being capable of forming fiber, and yellow represents the motif of being unable to form fiber. E) Representative TEM images of self‐assembly of NF‐1, NF‐2, and NF‐3 after incubation with recombinant furin (0.5 µg mL^−1^) at different times. F) HPLC analysis of NF‐1, NF‐1 with recombinant furin (0.5 µg mL^−1^), and NF‐3 for 12 h. G) Representative CLSM images of co‐localization of NF‐1_RhB_ (2 µm) with Golgi‐Tracker green in 4T1 cells after co‐culture for 8 h. Nucleus, blue (DAPI). H) Pearson's coefficient of CLSM images between NF‐1_RhB_ (2 µm) and different cell organelles, including the nucleus, endoplasmic reticulum, lysosome, mitochondria, and GA in 4T1 cells after 8 h of co‐culture. I) Representative TEM images of 4T1 cells incubated with PBS, NF‐1 (100 µm), and NF‐2 (100 µm) for 24 h, respectively. J) Immunofluorescence images of GM130 after PBS, NF‐1, or NF‐2 treatment in 4T1 cells. K) RUSH system analysis of the protein process and transportation in 4T1 cells after incubation of NF‐1 (100 µm) and NF‐2 (100 µm) for 24 h, respectively. L) Cell viability curves of 4T1 cells that were incubated with different peptides (NF‐1, NF‐2) at various concentrations (0, 20, 40, 80, 100, 200, 400, and 600 µm) for 48 h. M) Statistical results of apoptosis assay of 4T1 cells after incubation with PBS, NF‐1 (100 µm), and NF‐2 (100 µm) for 24 h. N) Diagram of GA dysfunction and apoptosis of tumor cells induced by tumor cell GA‐targeting self‐assembled peptide.

Although several studies have investigated GA‐targeted therapy,^[^ ^11^ ^]^ but the field is still in its infancy. For example, chondroitin sulfate can effectively target CD44 receptors on the tumor surface and its GA‐targeting capability has been confirmed, leading to a series of GA‐targeted nano‐delivery systems for effective treatments for both tumors and liver fibrosis.^[^ ^12^ ^]^ Recently, there has been significant interest in enzyme‐responsive peptides that self‐assemble in situ and disrupt subcellular organelles.^[^ ^13^ ^]^ However, modular designs, which allow each component to do its job, are still rare. Therefore, we focused on designing a modular self‐assembly peptide for tumor cell targeting, GA targeting, and GA destruction. It has been reported that furin exhibits high expression in the GA of cancer cells.^[^ ^14^ ^]^ As depicted in **Figure** **1**A, the expression level of furin is significantly higher in cancer cells (4T1 cells) compared to normal cells (L929 cells). Furthermore, enzyme‐linked immunosorbent assay (ELISA) results confirmed that the content of furin in 4T1 cells is approximately 12.8 times greater than that in L929 cells (Figure 1B). Additionally, immunofluorescence staining revealed a strong colocalization between furin and the tumor cell GA (Figure 1C). Consequently, we designed a self‐assembled peptide named NF‐1 with three functional modules arranged sequentially: 1) an Arg‐Gly‐Asp (RGD) module^[^ ^15^ ^]^ for hydrophilicity and tumor cell targeting; 2) an Arg‐Val‐Arg‐Arg (RVRR) module^[^ ^16^ ^]^ for hydrophilicity, furin responsiveness, and GA targeting; and finally, 3) a Lys‐Leu‐Val‐Phe‐Phe (KLVFF) module^[^ ^17^ ^]^ for hydrophobicity and amyloid‐like nano‐fibrous self‐assembly. To validate our design concept, two control peptides named NF‐2 and NF‐3 were also synthesized. In comparison to NF‐1, while NF‐2 can be cleaved by furin, it lacks the ability to self‐assemble into nano‐fibers due to the replacement of the KLVFF sequence with GGGGG. On the other hand, although NF‐3 possesses an amyloid‐like nano‐fibrous self‐assembling module consisting of a KLVFF sequence similar to those in NF‐1, it lacks tumor cell GA‐targeting capability. A schematic diagram of the chemical structure and assembly of the NF‐1 and its two control peptides (NF‐2 and NF‐3) in an aqueous solution is shown in Figure 1D.

The chemical structures of these peptides were confirmed using electrospray ionization mass spectrometry (ESI‐MS) and ^1^H nuclear magnetic resonance (^1^H NMR) (Figures S1A–C and S2A–C, Supporting Information). As depicted in Figure 1E, both NF‐1 and NF‐2 exhibited self‐assembled micellar morphologies, while NF‐3 displayed a nano‐fibrous morphology as observed by transmission electron microscopy (TEM). The critical aggregation concentrations (CAC) for NF‐1, NF‐2, and NF‐3 were determined to be 17.62, 14.93, and 11.69 µm respectively (Figure S3A–C, Supporting Information), indicating the excellent self‐assembly capabilities even under diluted conditions. The hydrodynamic particle size of NF‐1 in PBS (pH 7.4) with 10% FBS were monitored and showed minor change over 7 days, suggesting the good stability in simulated physiological environment (Figure S4, Supporting Information). Subsequently, the furin‐responsiveness of NF‐1 was evaluated in aqueous solution where it was found that upon incubation with furin (0.5 µg mL^−1^) for extended periods of time (6 and 24 h), the morphology progressively transformed into nano‐fibers similar to the self‐assembled morphology of NF‐3; whereas as a control experiment, the presence of furin did not alter the micellar morphology of NF‐2. Meanwhile, dynamic light scattering (DLS) analysis revealed that the mean hydrated diameter of NF‐1 exhibited a range from 141.8 to 825.4 nm in the presence of furin (Figure S5, Supporting Information), accompanied by corresponding changes in count rate at specific time points (Figure S6, Supporting Information). Conversely, there were minimal alterations observed in the count rate for NF‐2 upon the addition of furin. Circular dichroism (CD) and Fourier‐transform infrared spectroscopy (FTIR) spectra further corroborated the preservation of identical secondary structures between NF‐1 and NF‐3 following incubation with furin (Figure S7, Supporting Information). Moreover, high‐performance liquid chromatography (HPLC) analysis confirmed the furin‐responsive cleavage of NF‐1 into NF‐3 (Figure 1F). Collectively, these findings demonstrate that our designed NF‐1 exhibits remarkable responsiveness to furin, leading to a transformative self‐assembled morphology transition from micelles to nano‐fibers.

It is imperative to ascertain the selectivity of NF‐1′s self‐assembled morphological transformation on GA in cancer cells. To this end, NF‐1 was labeled with rhodamine B and then characterized by ESI‐MS, ^1^H NMR, and CAC (NF‐1_RhB_, Figures S1D–S3D, Supporting Information). Following an incubation period of 8 h with 4T1 cells, the intracellular content of NF‐1_RhB_ was nearly 3.13‐fold higher compared to L929 cells as confirmed by flow cytometry (FCM) analysis (Figure S8, Supporting Information). Furthermore, there was significant colocalization between NF‐1_RhB_ and GA‐tracker Green (Pearson's coefficient = 0.897), surpassing those observed for the nucleus (0.020), endoplasmic reticulum (0.219), lysosome (0.348), and mitochondria (0.361). This observation underscores the targeted affinity of NF‐1 toward tumor cell GA (Figure 1G,H; Figure S9, Supporting Information). The targeted accumulation of NF‐1 within tumor cell GA was further quantified based on fluorescence intensity measurements of NF‐1_RhB_ along with the volume occupied by GA within the cell (Figure S10, Supporting Information). When the extracellular incubation concentration of NF‐1_RhB_ was 15 or 30 µm, the concentration in GA was significantly higher at 1.76 or 7.18 mm, respectively, indicating enough concentration for NF‐1′ morphological transformation.

The fragmentation of GA in 4T1 cells was revealed following incubation with NF‐1 for 2 h and later by biological TEM images (Figure 1I; Figure S11, Supporting Information). Notably, NF‐2 lacking the nano‐fibrous module exhibited negligible impact on GA morphology after 24 h incubation. Additionally, immunofluorescence staining demonstrated down‐regulation of GM130,^[^ ^18^ ^]^ a structural protein associated with GA integrity, upon treatment with NF‐1 but not with NF‐2 for 24 h (Figure 1J). These findings were further corroborated by Western blot (WB) analysis (Figure S12, Supporting Information). Collectively, our results support the conclusion that tumor cell GA‐targeting NF‐1 can undergo morphological transformation from micelles into nano‐fibers within GA upon furin cleavage, resulting in selective fragmentation of GA. The primary function of the GA is widely recognized as protein processing, modification, and transportation. Therefore, the impact of selective fragmentation of tumor cell GA caused by NF‐1 on its biological function was investigated using a live‐cell retention system called RUSH (retention using selective hooks).^[^ ^19^ ^]^ This system enables visualization of intracellular protein processing and trafficking. Figure 1K demonstrates significant colocalization between Rush‐eqsm‐gfp (green) and DiD (red plasma membrane fluorescent probe) in both PBS‐treated and NF‐2‐treated 4T1 cells. However, after NF‐1 treatment, green fluorescence predominantly localizes to the GA. These findings indicate that NF‐1‐induced fragmentation of GA leads to dysfunction, thereby impairing protein processing and transportation.

To assess the effect of NF‐1‐induced GA fragmentation and dysfunction, 3‐(4,5‐dimethylthiazol‐2‐yl)‐2, 5‐diphenyl tetrazolium bromide (MTT) assay and apoptosis assay were firstly performed to evaluate cytotoxicity against different cell lines. As anticipated, NF‐1 exhibited negligible cytotoxicity toward L929 cells; however, it displayed an IC_50_ value of 122.1 µM against 4T1 cells after 48 h exposure. No cytotoxic effects were observed for NF‐2 against cancer cells (Figure 1L; Figure S13, Supporting Information). Furthermore, Annexin V‐FITC/PI assay revealed a high percentage of apoptosis exclusively in 4T1 cells treated with NF‐1 (Figure 1M; Figure S14, Supporting Information), confirming the selective cytotoxicity induced by NF‐1. The collective findings presented herein demonstrate that the self‐assembled peptide NF‐1, which specifically targets tumor cell GA, induces cell apoptosis as well as structural fragmentation and dysfunction of GA (Figure 1N).

### 2.2 Tumor Cell GA‐Targeting Self‐Assembled Peptide Reshapes Immunosuppressive Network for Effective Immunotherapy on Orthotopic BRCA Model

**Figure 2:**
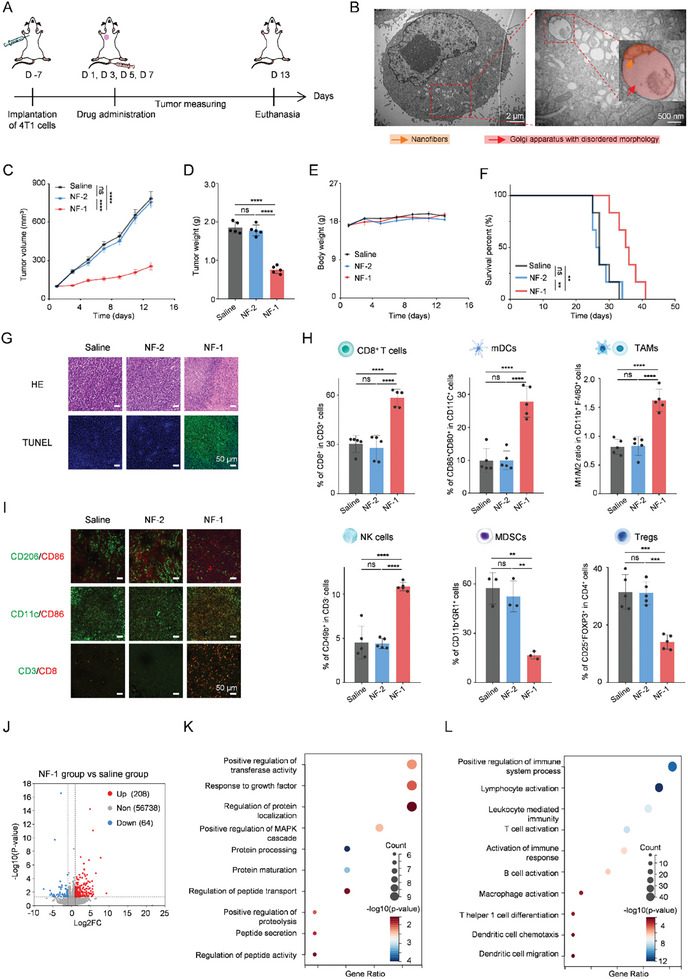
Tumor cell GA‐targeting self‐assembled peptide reshapes immunosuppressive network for effective immunotherapy on orthotopic BRCA model. A) Schematic illustration of the experimental timeline of tumor inhibition. B) TEM images of the GA structure in tumor cells after intravenous injection of NF‐1 (15 mg kg^−1^) for 24 h. C) Tumor volume and D) weight of isolated tumor tissues of mice after treatment of saline, NF‐2 (15 mg kg^−1^), and NF‐1 (15 mg kg^−1^) (*n* = 5). E) Change of mice's body weight during treatment processing (*n* = 5). F) Survival curves of mice after saline, NF‐1, and NF‐2 treatment. G) Representative images of H&E staining and TUNEL staining of isolated 4T1 tumor tissues at the end of treatment. H) Statistical results of flow cytometry of CD8^+^ T cells, mDCs, TAMs, NK cells, MDSCs, and Tregs after saline, NF‐1, and NF‐2 treatment. I) Immunofluorescence images of TAMs, mDCs, and CD8^+^ T cells after saline, NF‐1, and NF‐2 treatment. J) Volcano plot of differentially expressed genes in tumor tissues after saline and NF‐1 treatment. K) GO enrichment analysis of down‐regulated genes. L) GO enrichment analysis of up‐regulated genes. Statistical *p*‐values: ^**^ *p* < 0.01; ^***^ *p* < 0.001; ^****^ *p* < 0.0001; ns, no significance.

Considering that NF‐1 can induce tumor cell apoptosis as well as dysfunction of GA in vitro, it is necessary to investigate the tumor therapy effect of NF‐1 in vivo. A schematic illustration of the experimental timeline of tumor inhibition is shown in **Figure** **2**A. In 4T1 tumor‐bearing BALB/c mice, NF‐1 gradually accumulated at the tumor site and exhibited its highest fluorescence intensity 24 h post i.v. injection, indicating its effective tumor‐targeting ability (Figure S15, Supporting Information). The mice‐bearing tumors were then subjected to treatments with saline, NF‐2 (15 mg kg^−1^), and NF‐1 (15 mg kg^−1^). Subsequently, biological TEM imaging revealed GA fragmentation within the tumor cells at 24 h following NF‐1 administration, which can be attributed to in situ assembly into nano‐fibers (Figure 2B). As depicted in Figure 2C, both saline and NF‐2 treatments resulted in rapid tumor growth with volumes reaching ≈780 mm^3^ within a span of 13 days. Notably, treatment with NF‐1 significantly suppressed tumor volume (≈250 mm^3^). NF‐1 treatment demonstrated a remarkable inhibition ratio of tumors by 61.2% compared to the saline group in the analysis of extracted tumors’ weight and photographic images (Figure 2D; Figure S16, Supporting Information). Additionally, the negligible side effects of NF‐1 were demonstrated by assessing the body weight of mice (Figure 2E). Meanwhile, treatment with NF‐1 significantly prolonged mouse survival time compared to saline and NF‐2 (Figure 2F). Hematoxylin and eosin (H&E) staining of tumor tissues and terminal deoxynucleotidyl transferase‐mediated dUTP nicked labeling (TUNEL) assay further confirmed that compared to saline or NF‐2 groups, NF‐1 substantially increased apoptosis and necrosis rates among tumor cells (Figure 2G). H&E staining on major organs was conducted (Figure S17, Supporting Information), and the results showed that NF‐1 and NF‐2 did not cause toxic effects on major organs. Additionally, both NF‐1 (100 µm) and NF‐2 (100 µm) exhibited minimal hemolysis, with levels consistently below 5% (Figure S18, Supporting Information). Collectively, NF‐1 exhibited superior antitumor efficacy without inducing toxic side effects on the orthotopic 4T1 tumor‐bearing model.

To explore the reasons for the superior therapeutic effect of NF‐1 treatment, immune‐related analyses were performed on samples collected at the end of treatment. As depicted in Figure 2H, NF‐2 treatment had no effect on the infiltration levels of immune cells (Figure S19, Supporting Information). However, compared to the saline group, treatment with NF‐1 resulted in a significant increase in the infiltration level of immune cells, including CD8^+^ T cells (≈1.9‐fold), mDCs (≈2.8‐fold), M1/M2 macrophage ratio (≈2.0‐fold), and NK cells (≈2.4‐fold). On the contrary, NF‐1 treatment reduced the activation rate of MDSCs (from 57.33% ± 9.56% to 16.63% ± 2.36%) and Tregs (from 31.50% ± 6.02% to 14.12% ± 2.49%). Furthermore, NF‐1 treatment promoted the activation rate of Memory T cells from 8.18% ± 2.36% to 20.20% ± 2.26% (Figure S20A, Supporting Information). The levels of IFN‐γ (≈1.5‐fold higher than the saline group) and TNF‐α (≈2.6‐fold higher) were also found to be significantly elevated following NF‐1 treatment (Figure S20B,C, Supporting Information). Immunofluorescence results further demonstrated that NF‐1 treatment effectively stimulated the activation of mDCs and CD8^+^ T cells, leading to an enhanced M1/M2 ratio (Figure 2I). Furthermore, RNA sequencing was conducted on tumor tissues following various treatments. As depicted in Figure 2J, after NF‐1 treatment, 208 genes were up‐regulated while 64 genes were down‐regulated when compared with saline treatment. KEGG and GO analysis (Figure 2K,L; Figures S21 and S22, Supporting Information) revealed that the up‐regulated genes following NF‐1 treatment were enriched in positive regulation of immune system processes such as “Lymphocyte activation”, “T cell activation”, “B cell activation”, “Macrophage activation”, and “Dendritic cell chemotaxis”. Meanwhile, the down‐regulated genes were enriched in “Positive regulation of transferase activity”, “Response to growth factor”, and “Positive regulation of protein localization”. These RNA sequencing results align with both in vitro and in vivo experimental findings. These results suggest that treatment of NF‐1 can regulate the infiltration of multiple immune cells by targeting and intervening in the tumor cell GA, thereby reshaping the immunosuppressive network for effective immunotherapy. These findings also suggest that the tumor cell GA may mediate the formation of an immunosuppressive network. However, the mechanism remains to be investigated.

### 2.3 Tumor Cell‐Derived GA‐Dependent MIF Mediates the Formation of Immunosuppressive Network

**Figure 3:**
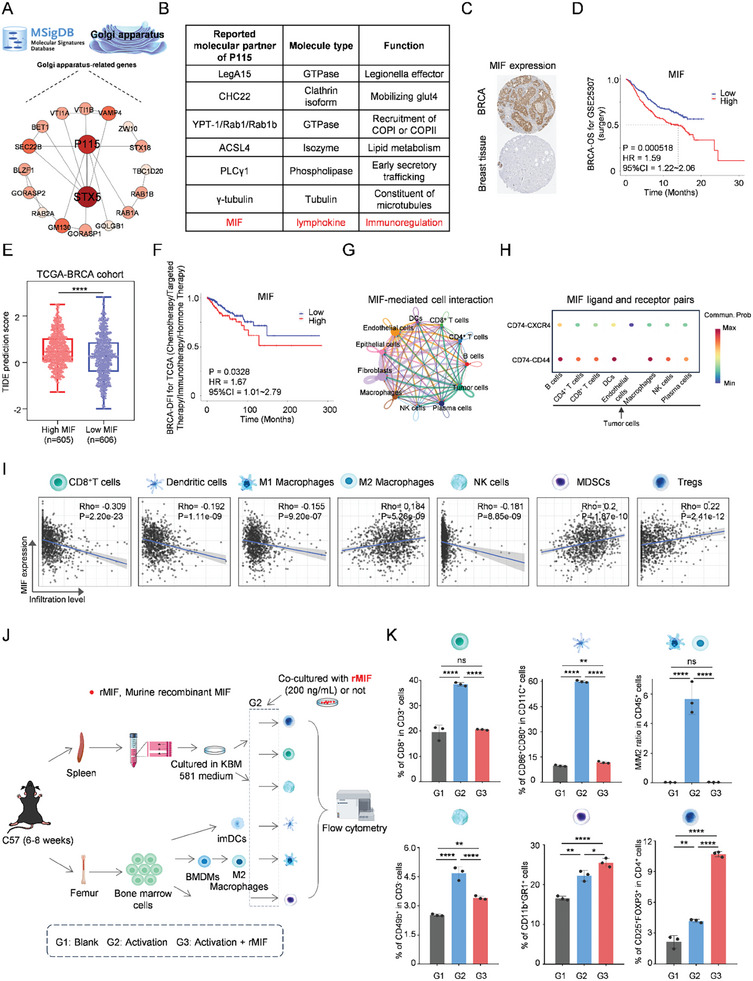
Tumor cell‐derived GA‐dependent MIF mediates the formation of an immunosuppressive network. A) PPI network of GA‐related genes. B) MIF was screened as an immune‐related regulator of the reported molecular partner of P115. C) MIF protein expression in BRCA and normal breast tissue in the HPA database. D) Survival curve of OS with high‐ and low‐expression of MIF in BRCA for GSE25037. E) Comparison of immunotherapy predictions between high‐ and low‐ expression of MIF according to the TIDE algorithm in the TCGA‐BRCA cohort. F) Survival curve of DFI after chemotherapy/targeted therapy/immunotherapy/hormone therapy with high‐ and low‐expression of MIF in BRCA. G) MIF‐mediated cell interaction from GSE176068. H) Showing of MIF ligand and receptor pairs (L‐R pairs), with tumor cells as sender and other cell types as receiver from GSE176068. I) Correlation between MIF expression and immune cells in BRCA based on the TIMER database. J) Flow chart of primary cell extraction and culture of multiple immune cells, cytokine‐stimulated differentiation, and exogenous rMIF intervention in immune cell differentiation. K) Flow cytometry statistical results of different immune cell differentiation outcomes induced by different cytokines and rMIF intervention.

To explore the formation of an immunosuppressive network of BRCA mediated by tumor cell GA, we performed multiple omics analyses comprising bulk RNA sequencing data, spatial transcriptome sequencing (ST‐seq), and single‐cell RNA‐sequencing data (scRNA‐seq). Firstly, the gene set “GOBP_GOLGI_ORGANIZATION” consists of 157 GA‐related genes was downloaded from the Molecular Signatures Database (MSigDB) and a protein‐protein interaction (PPI) network of these genes was visualized using the STRING database, and Cytoscape software while the confidence of minimum required interaction score means 0.99. As a result in **Figure** **3**A, STX5, and P115 were the key genes of Golgi‐related genes. By exploring the two genes in the GeneCards database, STX5 mainly mediates endoplasmic reticulum to GA transport and maintains the stacked and inter‐connected structure of the GA.^[^ ^20^ ^]^ P115 is a peripheral membrane protein that is required for transcytotic fusion and/or subsequent binding of the vesicles to the target membrane and is responsible for the transport of the GA to the exocrine cell.^[^ ^21^ ^]^ Considering that we are more concerned about the formation of immunosuppressive networks, P115 was screened for further analysis. To explore downstream molecules that might bind to P115 and play an important role in TIME reshaping, we searched PubMed, Web of Science, and Scopus and collected original studies to identify molecular partners of P115 that have been discovered so far.^[^ ^22^ ^]^ As shown in Figure 3B, MIF is the most suitable candidate molecule.

In BRCA tissues, MIF expression demonstrated “high” staining with “strong” intensity in both cytoplasmic and membrane domains; conversely, normal breast tissues showed no detectable staining with negative intensity from the human protein atlas (HPA) database (Figure 3C). Immunohistochemistry analysis revealed up‐regulated expression of both MIF and P115 in tumor tissues compared with para‐carcinoma tissues from BRCA patients (Figure S23, Supporting Information). P115 was also found to be highly expressed in BRCA according to the HPA database (Figure S24, Supporting Information). Kaplan–Meier (KM) survival plot revealed that higher MIF expression levels were associated with shorter overall survival time among surgery‐treated patients from the GSE25307 cohort (HR = 1.59; CI = 1.22–2.06; p = 0.0005) (Figure 3D). The role of MIF in the prediction of immunotherapy effects and prognosis was further investigated. A comparison of tumor immune dysfunction and exclusion (TIDE) scores^[^ ^23^ ^]^ was performed in the TCGA‐BRCA cohort to assess the expression levels of MIF between patients with high and low expression (Figure 3E). The findings revealed that patients with high MIF expression exhibited elevated TIDE scores, indicating a reduced sensitivity to immunotherapy. Additionally, KM survival analysis demonstrated that higher MIF expression in BRCA was associated with a shorter disease‐free interval (DFI) time (HR, 1.67; CI = 1.01–2.79; p = 0.0328) among chemotherapy/targeted therapy/immunotherapy/hormone therapy‐treated patients from the TCGA‐BRCA cohort (Figure 3F). An integrated analysis of scRNA‐seq and ST‐seq data further revealed distinct tumor microenvironments, characterized by a notable presence of immune cells such as CD8^+^ T cells and CD4^+^ T cells predominantly in non‐tumor regions, with a significant reduction observed within tumor areas in TNBC (Figure S25A, Supporting Information). Moreover, MIF exhibited high expression levels in the tumor tissues (Figure S25B, Supporting Information). Multiple scRNA‐seq datasets consistently demonstrated higher MIF expression in tumor cells compared to immune cells and stromal cells (Figure S25C, Supporting Information). By employing CellChat analysis, an extensive and intricate MIF‐mediated intercellular communication network was revealed in BRCA (Figure 3G). Furthermore, our findings illustrated the interaction strength between MIF ligand‐receptor pairs (“CD74‐CD44” vs “CD74‐CXCR4”), with tumor cells acting as senders while immune cells served as receivers for these signals (Figure 3H). Spatial feature plots were utilized to demonstrate the expression of MIF, CD74, CXCR4, and CD44 in tissue sections using ST‐seq (Figure S25D, Supporting Information). ScRNA‐seq data also indicated high expression of MIF across almost all cell types (Figure S25E, Supporting Information). The predominant source of MIF signals was identified as tumor cells, while immune cells were found to receive these signals, suggesting that tumor cell‐derived MIF may mediate the formation of the immunosuppressive network (Figure S25F, Supporting Information). Furthermore, the relationships between MIF expression and genetic alterations were explored by analyzing mutated genes in 1092 cases of BRCA obtained from the TCGA database (Figure S25G, Supporting Information). In conclusion, these genes are intricately associated with immunotherapy across various cancers, thereby reinforcing our assertion that MIF may influence the efficacy of immunotherapy. Furthermore, the associations between MIF expression and immune checkpoint expression across various cancers were analyzed based on TCGA cohorts (Figure S26, Supporting Information). A noteworthy inverse relationship was observed between MIF level and the expression of CD274 (PD‐L1), BTLA, CTLA4, TIGIT, HAVCR2 (TIM‐3), IDO1, CD28, IFNG, CD80, ICOS, and TNF. To further explore whether tumor cell‐derived GA‐dependent MIF mediates the formation of the immunosuppressive network by regulating multiple types of immune cells, we conducted an analysis of the correlation between MIF expression and immune cell infiltration in the TCGA‐BRCA cohort (Figure 3I). The results revealed significant negative correlations between MIF and infiltrating levels of CD8^+^ T cells (r = ‐0.309, p = 2.20e‐23), Dendritic cells (r = ‐0.192, p = 1.11e‐09), M1 Macrophages (r = ‐0.155, p = 9.20e‐07), and NK cells (r = ‐0.181, p = 8.85e‐09). Conversely, there were significant positive correlations between MIF and infiltrating levels of M2 Macrophages (r = 0.184, p = 5.26e‐09), MDSCs (r = 0.2, p = 1.87e‐10), and Tregs (r = 0.22, p = 2.41e‐12).

By integrating multiple omics data, we propose a hypothesis suggesting that tumor cell‐derived GA‐dependent MIF may regulate multiple types of immune cells including CD8^+^ T cells, DCs, M1 macrophages, NK cells, M2 macrophages, MDSCs and Tregs to mediate the formation of immunosuppressive network in BRCA, leading to diminished efficacy of immunotherapy. In order to validate our hypothesis, preliminary verification was conducted through in vitro cellular experiments. The extraction, cultivation, and cytokine‐stimulated differentiation methods for various immune cells have been previously reported.^[^ ^24^ ^]^ Based on these methods, experiments were performed as depicted in Figure 3J. During the final step of stimulating the differentiation of different immune cells, murine recombinant MIF (rMIF) was added at a concentration of 200 ng mL^−1^ for co‐culture to simulate the regulatory effect exerted by tumor cell‐derived MIF on immune cells within the tumor microenvironment. Subsequently, FCM analysis was conducted. Representative FCM data along with corresponding statistical results were presented for CD8^+^ T cells, mDCs, M1/M2 macrophage ratio, NK cells, MDSCs, and Tregs (Figure 3K; Figure S27, Supporting Information). In comparison to the blank group, the corresponding cytokine‐stimulated immune cells exhibited obvious activation and differentiation. Compared with only cytokine stimulation group, coculture with rMIF resulted in a significant decrease in the activation rate of CD8^+^ T cells from 38.40% ± 0.70% to 20.53% ± 0.21%, mDCs (from 59.83% ± 0.61% to 11.73% ± 0.59%), M1/M2 macrophage ratio (from 5.67 ± 1.06 to 0.04 ± 0.01), and NK cells (from 4.69% ± 0.34% to 3.41% ± 0.10%), even approached to that of the blank group. Moreover, it further facilitated the activation rate of MDSCs (from 22.20% ± 1.28% to 25.47% ± 1.10%) and Tregs (from 4.18% ± 0.16% to 10.70% ± 0.27%). The results suggest that treatment with rMIF suppresses the activation of multiple immune‐promoting cells while promoting the activation of multiple immune‐suppressive cells, which aligns with the conclusions drawn from the aforementioned multi‐omics analysis. By integrating bioinformatics analysis and experimental verification, we found that tumor cell‐derived GA‐dependent MIF mediates the formation of immunosuppressive network by regulating multiple types of immune cells.

### 2.4 Tumor Cell GA‐Targeting Self‐Assembled Peptide Reshapes MIF‐Mediated Immunosuppressive Network

**Figure 4:**
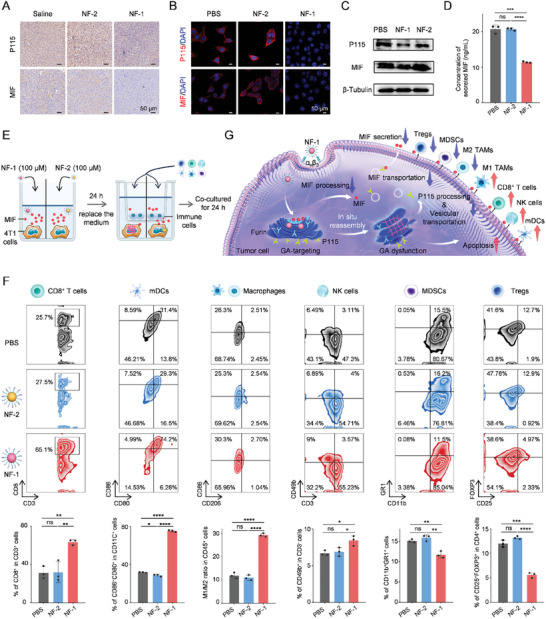
Tumor cell GA‐targeting self‐assembled peptide reshapes MIF‐mediated immunosuppressive network. A) Immunohistochemical staining of P115 and MIF after saline, NF‐1, or NF‐2 treatment in the 4T1 tumor‐bearing model. B) Representative immunofluorescence images and C) WB assay of P115 and MIF after PBS, NF‐1, or NF‐2 treatment in 4T1 cells. D) ELISA analysis of secreted MIF of 4T1 cells in the supernatant after different treatments. E) Schematic diagram of co‐culture of 4T1 cells which were treated with peptides to reduce 4T1 cells‐derived MIF or not with different immune cells. F) Representative flow cytometry and corresponding statistical results of CD8^+^ T cells, mDCs, Macrophages, NK cells, MDSCs, and Tregs. G) Schematic diagram of tumor cell apoptosis and MIF‐mediated immunosuppressive network reshaping induced by tumor cell GA‐targeting self‐assembled peptide NF‐1. Statistical *p*‐values: ^*^ *p* < 0.05; ^**^ *p* < 0.01; ^***^ *p* < 0.001; ^****^ *p* < 0.0001; ns, no significance.

To investigate whether tumor cell GA‐targeting self‐assembled peptide NF‐1 can reshape the MIF‐mediated immunosuppressive network, we performed the following experiments. First, NF‐1 treatment led to a noticeable reduction in the expression levels of both P115 and MIF compared to the saline and NF‐2 treatment in the 4T1 tumor‐bearing model (**Figure** **4**A). Consistent results were observed in 4T1 cells in vitro (Figure 4B). Consistent with the immunofluorescence results, WB analysis also demonstrates a significant decrease in both P115 and MIF following NF‐1 treatment when compared to the control (Figure 4C). These results indicate that NF‐1 treatment effectively suppresses the synthesis of endogenous MIF by tumor cells. Moreover, the level of MIF secretion in the medium after NF‐1 treatment decreased to approximately half of that observed in the control group (from 20.77 to 11.42 ng mL^−1^). However, there was no change in MIF secretion after NF‐2 treatment (20.73 ng mL^−1^) (Figure 4D).

To explore whether multiple immune cells in an immunosuppressive network can be reprogrammed after NF‐1 treatment‐induced MIF reduction. The following experiment was conducted and the schematic diagram in Figure 4E illustrates the co‐culture of 4T1 cells with different immune cells, wherein NF‐1 or Nf‐2 treatment is applied to disturb the production of MIF derived from 4T1 cells. As a result (Figure 4F), The treatment of NF‐2 has minimal impact on the activation or inhibition of immune cells. Regarding the treatment of NF‐1, it significantly augmented the activation level of immune cells, including CD8^+^ T cells (≈2.0‐fold), mDCs (≈2.3‐fold), M1/M2 ratio of TAMs (≈2.4‐fold), and NK cells (≈1.3‐fold) compared to the blank controls (Figure 4F). Additionally, there was an ≈1.5‐fold increase observed in the production of IL‐12 by mDCs, as well as enhanced secretion of TNF‐α by CD8^+^ T cells and M1 macrophages, when compared to the control group (Figure S28, Supporting Information). Conversely, NF‐1 treatment decreases the activation rate of MDSCs (from 15.07% ± 0.40% to 11.70% ± 0.82%) and Tregs (from 11.97% ± 0.70% to 5.57% ± 0.53%). All these findings demonstrate that NF‐1 treatment can reshape the MIF‐mediated immunosuppressive network by regulating multiple types of immune cells (Figure 4G). After endocytosis by the αvβ3 receptor on the cell membrane, tumor cell GA‐targeting NF‐1 further targets the GA and in situ reassembly in response to furin to destroy the GA. Firstly, apoptosis is induced. Second, P115 processing and vesicular transportation are weakened. Furthermore, MIF processing, transportation, and secretion are decreased. Eventually, the MIF‐mediated immunosuppressive network is reshaped and anti‐tumor immunity is activated. Through in vitro and in vivo experiments and multi‐omics analysis, the conclusion that NF‐1 treatment can reshape tumor cell‐derived GA‐dependent MIF‐mediated immunosuppressive network formation was validated in BRCA.

### 2.5 Reshaping MIF‐Mediated Immunosuppressive Network Enhances ICB Efficacy on CT26 Tumor‐Bearing Model

**Figure 5:**
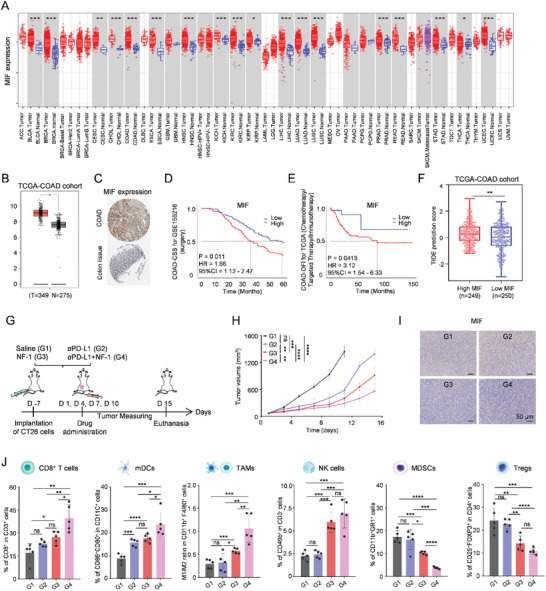
Reshaping the MIF‐mediated immunosuppressive network enhances ICB efficacy on the CT26 tumor‐bearing model. A) A pan‐cancer analysis of MIF expression with comparisons between tumor and normal tissues from the TIMER database. B) Boxplot analysis of the expression of MIF in tumor tissues and para‐carcinoma tissues from COAD patients in the TCGA‐COAD cohort. C) MIF protein expression in COAD and normal colon tissue in the HPA database. Survival curve of D) OS after surgery and E) DFI after chemotherapy/targeted therapy/immunotherapy with high‐ and low‐expression of MIF in COAD. F) Comparison of immunotherapy predictions between high‐ and low‐ expression of MIF according to the TIDE algorithm in the TCGA‐COAD cohort. G) Schematic illustration of the experimental timeline of tumor inhibition. H) Tumor volume of mice after treatment of saline (G1), αPD‐L1 (G2), NF‐1 (G3, 15 mg kg^−1^), and αPD‐L1 + NF‐1 (G4) (*n* = 5). I) Immunohistochemical staining of the expression of MIF at the end of treatment. J) Statistical results of flow cytometry of CD8^+^ T cells, mDCs, TAMs, NK cells, MDSCs, and Tregs after different treatments. Statistical *p*‐values: ^*^ *p* < 0.05; ^**^ *p* < 0.01; ^***^ *p* < 0.001; ^****^ *p* < 0.0001; ns, no significance.

To explore whether tumor cell‐derived GA‐dependent MIF could also be a therapeutic target in other tumor types, a pan‐cancer analysis was first performed. MIF was found to be upregulated in most cancer types (**Figure** **5**A). To verify the universality of our proposed treatment strategy, we performed the following validation in another high‐incidence tumor. Colorectal adenocarcinoma (COAD), which originates from epithelial cells, is commonly regarded as a “cold” tumor that is characterized by poor response to ICB therapy. First, a high expression level of MIF was observed in COAD (Figure 5B,C). Additionally, KM survival analysis revealed that higher MIF expression in COAD patients who underwent surgery was associated with shorter CSS time (HR = 1.66; CI = 1.12–2.47; p = 0.011) based on data from GSE159216 (Figure 5D). Higher MIF expression in COAD was significantly associated with shorter DFI time (HR, 3.12; CI, 1.54–6.33; p = 0.0413) in chemotherapy/targeted therapy/immunotherapy‐treated patients from the TCGA‐COAD cohort (Figure 5E). Moreover, analysis of TIDE scores revealed that patients with high MIF expression exhibited elevated TIDE scores within the TCGA‐COAD cohort (Figure 5F), indicating that tumor cell‐derived GA‐dependent MIF also contributes to the formation of immunosuppressive network in COAD, thereby compromising the efficacy of immunotherapy.

At the cellular experimental level, treatment with NF‐1 can also induce apoptosis and suppress GA‐dependent MIF expression and secretion in CT26 cells (Figure S29; Figures S30–S32, Supporting Information). Subsequently, to investigate the potential enhancement of ICB efficacy, we conducted a combination therapy of NF‐1 and PD‐L1 antibody (αPD‐L1) on the CT26 tumor‐bearing model. The experimental design is schematically illustrated in Figure 5G. As shown in Figure 5H, αPD‐L1 treatment alone failed to reduce tumor volume, and NF‐1 treatment exhibited superior tumor suppression compared to αPD‐L1 treatment alone, while the combined administration of NF‐1 significantly improved the therapeutic efficacy of αPD‐L1. H&E staining and TUNEL assay further confirmed that the combination therapy induced extensive necrosis in tumor cells (Figures S33 and S34, Supporting Information). Compared with saline treatment, αPD‐L1 treatment had little effect on the expression of MIF, while NF‐1 treatment alone or combined administration of NF‐1 significantly reduced the expression of MIF (Figure 5I; Figure S35, Supporting Information). Collectively, these findings highlight the enhanced efficacy of ICB achieved by utilizing tumor cell GA‐targeting self‐assembled peptide. To investigate the ability of NF‐1 to reshape the MIF‐mediated immunosuppressive network in COAD, changes in immune‐activated and immune‐suppressive cells were assessed. As depicted in Figure 5J and Figures S36 and S37 (Supporting Information), treatment with αPD‐L1 minimally impacted the level of immune cell infiltration within COAD. Conversely, NF‐1 treatment enhanced the activation rate of CD8^+^ T cells, mDCs, M1/M2 ratio of TAMs, NK cells, and Memory T cells while reducing the activation rate of MDSCs and Tregs. Furthermore, in comparison to αPD‐L1 treatment alone, the combination of αPD‐L1 and NF‐1 treatment exhibited an augmentation in the activation rate of CD8^+^ T cells (≈1.7‐fold), mDCs (≈1.5‐fold), M1/M2 ratio of TAMs (≈3.3‐fold), NK cells (≈2.8‐fold), and Memory T cells (≈4.5‐fold). On the other hand, the combination of αPD‐L1 and NF‐1 treatment reduced the activation rate of MDSCs (from 16.03% ± 4.33% to 3.69% ± 0.47%) and Tregs (from 22.68% ± 2.23% to 10.79% ± 1.32%). In addition, the combination treatment resulted in higher levels of TNF‐α and IFN‐γ compared to the single treatment (Figure S38, Supporting Information). Overall, NF‐1 treatment can enhance the ICB efficacy by reshaping the immunosuppressive network in COAD, converting a “cold” tumor into a “hot” tumor through the inhibition of tumor cell‐derived GA‐dependent MIF. Furthermore, the evaluation of body weight, H&E staining of major organs, and blood biochemical analysis in mice demonstrate the minimal systemic toxicity associated with our meticulously designed peptide (Figures S39 and S40, Supporting Information).

## 3 Conclusion

In summary, we have tailored a tumor cell GA‐targeting self‐assembled peptide (NF‐1) for effective immunotherapy via reshaping the MIF‐mediated immunosuppressive network. First, in vitro experiments demonstrate that NF‐1 can selectively and effectively disrupt tumor cell GA and induce tumor cell apoptosis. At the same time, the administration of NF‐1 was observed to reshape the immunosuppressive network for effective immunotherapy on the orthotopic BRCA model. Notably, we found that tumor cell‐derived GA‐dependent MIF mediates the formation of the immunosuppressive network through multi‐omics analysis and in vitro experiments. Furthermore, NF‐1 administration can convert a “cold” tumor into a “hot” tumor by increasing the infiltration level of CD8^+^ T cells, mDCs, NK cells, M1 macrophages, and memory T cells and reducing the activation rate of MDSCs and Tregs, thus enabling immunotherapy in BRCA and enhancing the ICB efficacy in COAD. Overall, the proposed self‐assembled peptide strategy of interfering tumor GA will provide different immunotherapy ideas for BRCA, COAD, and other cancers characterized by a “cold” immune microenvironment. Given the critical role of GA in both normal physiological functions and in promoting the formation of a tumor immunosuppressive network, exploring strategies to selectively inhibit the immunosuppressive function of tumor cell GA represents a promising research direction.

## 4 Experimental Section

### Peptide Synthesis and Characterization

NF‐1 and its analogs (NF‐2 and NF‐3) were synthesized based on the standard solid‐phase peptide synthesis protocol with the standard Fmoc strategy on the 2‐chlorotrityl resin. Briefly, 2‐chlorotrityl resin (1 equivalent) was swelled in dry N, N‐dimethylformamide (DMF) for 30 min, and then the first amino acid (2 equivalents) and N, N‐Diisopropylethylamine (DIEA, 4 equivalents) were loaded onto the resin solution and shaken under room temperature for 3 h. After loading the first amino acid to the resin, the capping regent (400 µL methanol in 4 mL DMF) was used to cap all the active sites of the resin for an additional 30 min. Fmoc group was then removed with 20% piperidine in DMF; the next Fmoc‐protected amino acid was coupled to the free amino group using HBTU (2 equivalents), HOBT (2 equivalents), and DIEA (4 equivalents) as the coupling reagent. Repeat the previous step until the synthesis of the peptide is complete. The N‐terminus of peptides (NF‐1 = NF‐3) were capped as acetamides using acetic anhydride (6 equivalents) for 3 h at room temperature. The crude compound NF‐1 = NF‐3 was obtained after cleaving the peptide from the resin by trifluoroacetic acid (TFA)/triisopropylsilane/water (95:2.5:2.5 v/v) for 3 h and then purified by reverse phase HPLC with double‐distilled water (ddH_2_O) containing 0.1% TFA and MeOH containing 0.1% TFA. 10 mg purified peptide was dissolved in deuterated reagent methanol or DMSO and then characterized by 1H NMR using Bruker AVANCE III 400 MHz spectrometer. ESI‐MS (m/z): Calcd. for C_72_H_119_N_25_O_16_ (NF‐1), [M+2H]^2+^, 795.97, [M+3H]^3+^, 530.98, [M+4H]^4+^, 398.48, Found, 796.30, 531.20, 398.60; Calcd. for C_47_H_84_N_24_O_16_ (NF‐2), [M+2H]^2+^, 621.32, [M+3H]^3+^, 414.55, [M+4H]^4+^, 311.16, Found, 621.05, 414.55, 310.75; Calcd. For C_35_H_52_N_6_O_6_ (NF‐3), [M+H]^+^, 653.39, Found, 653.20. It is worth mentioning that Fmoc‐Lys (Boc)‐OH in NF‐1_RhB_ has been replaced with Fmoc‐Lys (Dde)‐OH to make it easier to conjugate rhodamine B (RhB). This detail is as follows. After synthesis of all the amino acids and acetic anhydride on the 2‐chlorotributyl resin, the resin was soaked in a solution of DMF containing 2% hydrazine for 10 min. The solution was removed, and the above operation was repeated three times. Finally, the partially protected peptide resin was washed several times with DMF. Then, RhB solution (2 equivalents) was added to the resin for 6 h. The rest of the process is identical to the peptide synthesis steps and will not be repeated here. ESI‐MS (m/z): Calcd. for C_100_H_148_N_27_O_18_Cl (NF‐1_RhB_), [M+2H]^2+^, 1008.58, [M+3H]^3+^, 672.72, [M+4H]^4+^, 504.79, Found, 1008.95, 672.40, 504.70.

### Preparation of Peptide Self‐Assemblies

The purified peptides were dissolved in ddH_2_O as the stock solution (8 mm). The stock solutions were subsequently stored at 4 °C overnight to achieve as much balance as possible. The self‐assembled peptides for the following experiments with various concentrations will not be repeated here.

### Characterization of Peptide Self‐Assemblies—*Morphological Characterization*

TEM specimens were prepared by depositing the individual peptide aqueous solution (2 µL) onto a carbon‐coated copper grid and dried entirely under ambient conditions. Structural information of series peptides was observed operating at 200 kV. The data were analyzed using Digital Micrograph software characterizations of structure. Freeze‐dried NF‐1 or other self‐assembled peptides (2.0 mg) were crushed together with potassium bromide crystals for FTIR analysis. The spectra were scanned 32 times from 4000 to 400 cm^−1^. CD spectra for the solution samples were obtained in ddH_2_O using a 0.1 mm quartz slip cuvette with cover. CD spectra were collected over the range of 190–260 nm with a step of 0.5 nm, bandwidth of 1 nm, and collection time of 2 s per step under nitrogen atmosphere at room temperature, taking three averages. The CAC of amphiphilic peptides was examined according to the excitation spectral change utilizing pyrene as a hydrophobic fluorescent probe. Aliquots of pyrene solutions (3.6 µm in acetone, 3 mL) were added to tubes, and acetone was allowed to evaporate. Then, 0.8 mL aqueous solution (10 mm PB, pH 7.40) of amphiphilic peptide with a concentration ranging from 0.2 to 100 µm was added to the container. After shaking slightly for 4 h at 37 °C, the solution stayed overnight in a thermostatic water bath (37 °C), reaching the dissolution equilibrium of pyrene. Emission was carried out at 390 nm, and excitation spectra were recorded ranging from 300 to 360 nm.

### Co‐Localization of Subcellular Organelles with NF‐1_RhB_

First, 4T1 cells were seeded in a 24‐well plate containing 14 mm cover glass (10^4^ cells per well) overnight, and unadhered cells were removed using PBS. The cells were then incubated with NF‐1_RhB_ (2 µm) for 8 h. The cells were then washed three times carefully using PBS, followed by the addition of commercial Golgi‐tracker green (0.28 mm) and incubation for 30 min under 4 °C. After washing three times using PBS, those cells continued growing and incubating for another 30 min under 37 °C. Later, the cells were washed with PBS three times, fixed with 4% PFA for 20 min, and stained with DAPI (10 µg mL^−1^) for 10 min and subsequently, visualized by CLSM (N‐SIM/N‐STORM) at 550 nm excitation for NF‐1_RhB_, 488 nm excitation for Golgi‐Tracer Green, and 405 nm excitation for DAPI, respectively. The cells were then washed three times carefully using PBS, followed by adding commercial Mito‐Tracer Green (50 nm) and incubating for another 30 min under 37 °C. Later, the cells were washed with PBS three times, fixed with 4% PFA for 20 min, and stained with DAPI (10 µg mL^−1^) for 10 min and subsequently, visualized by CLSM (LSM880) at 550 nm excitation for NF‐1_RhB_ B, 488 nm excitation for Mito‐Tracer Green, and 405 nm excitation for DAPI, respectively. The same experiment protocol was also applied in endoplasmic reticulum imaging with ER‐Tracer Green (1 µm) and lysosome imaging with Lyso‐Tracer Green (50 nm).

### Immunofluorescence Assay

The cells were then washed three times carefully using PBS, fixed with 4% PFA for 20 min, and incubated with anti‐P115 (1:1000) or anti‐MIF (1:1000) for 1 h under 37 °C. Later, the cells were washed with PBS and incubated with Alexa Fluor 488‐labeled Goat Anti‐Rabbit IgG(H+L) (Yeasen Biotech Co., Ltd., #33106ES60, 1:100) or Alexa Fluor 594‐labeled Goat Anti‐Rabbit IgG(H+L) (Yeasen Biotech, #33112ES60, 1:100) for 2 h. Finally, the cells were stained with DAPI (10 µg mL^−1^) for 10 min and visualized by CLSM (LSM880).

### Western Blot

4T1 or CT26 cells were seeded into a 6‐well plate (NEST Biotechnology Co., Ltd) at the density of 2 × 10^5^ cells. The cells are treated differently and then lysed in RIPA lysis buffer on ice for 30 min. They were then collected and then conducted with centrifugation using 12 000 rpm for 15 min at 4 °C. Protein concentrations were measured using protein assay reagents, and equal amounts of protein per lane were separated on SDS‐PAGE gel and transferred to a PVDF membrane. First, the membranes were sealed with 10 mL 5% BSA for 2 h. Then, the membranes were washed with TBST three times for 10 min each and incubated with anti‐P115 (ABclonal Biotechnology, #A20950, 1:1000), anti‐MIF (ABclonal Biotechnology, #A1391, 1:1000), anti‐furin (Affinity Biosciences, #DF13231, 1:1000), aiti‐GM130 (Abmart Bio‐tech Co. Ltd., Shanghai, China, #T55142, 1:1000), anti‐β‐tublin (Beyotime Biotechnology, #AF2835, 1:1000), and anti‐GAPDH (Affinity Biosciences, #AF7021, 1:2000) primary antibodies at 4 °C overnight, respectively. After incubation, the membranes were washed with TBST thrice for 10 min each and incubated with HRP‐goat anti‐rabbit IgG (H+L) (UpingBio Technology, # YP848537, 1:1000) for 2 h. Finally, WB was visualized by an enhanced chemiluminescence detection system. The same protocol was performed for CT26 cells.

### Flow Cytometry

Cells or mouse tissue were prepared into a single‐cell suspension for flow cytometry (FACSCantoII, BD) analysis. FITC‐CD11c (#117 305), APC‐CD80 (#104 714), PE‐CD86 (#159 204), APC‐CD3 (#100 204), PE‐CD4 (#100 407), FITC‐CD8a (#100 705), APC‐GR1 (#108 411), Alexa Fluor 700‐CD45 (#147 716), PE‐CD11b (#101 205), PE‐Foxp3 (#126 403), APC‐CD206 (#141 707), PE‐CD49b (#103 506), FITC‐CD25 (#101 908), PE‐CD62L (#161 204), and percp/cy5.5‐CD44 (#103 031) were purchased from BioLegend, Inc. (San Diego, USA). Annexin V‐FITC Apoptosis Detection Kit (#CA1020) was purchased at Beijing Solarbio Science &Technology Co., Ltd. All reagents were used according to the manufacturer's specifications and the data were analyzed using FlowJo software (version 10.7.1).

### ELISA Assay

Cell supernatant or tissue abrasive solution was collected for ELISA assay, including murine TNF‐α (NeoBioscience Technology Co. Ltd.), murine MIF (Beijing Dogesce), murine furin, IL‐12 and FIN‐γ (Jiangsu Meimian Industrial Co., Ltd.)

### Primary Immune Cells Generation and Stimulation

Primary cells were all extracted from femurs or spleens from 6–8 weeks C57BL/6 mouse strains. The femur of C57BL/6 mice was isolated aseptically, and the bone marrow cells were removed and lysed with erythrocyte lysate to obtain bone marrow mononuclear cells, which were cultured in 6‐well plates with freshly prepared DMEM containing IL‐4 (10 ng mL^−1^) (MedChemExpress, #HY‐P70644) and GM‐CSF (10 ng mL^−1^) (PeproTech, #315‐03) for 7 d. The suspension cells (immature DCs) and adherent cells (BMDM cells) were isolated, and BMDM cells were continued to be treated with a whole culture containing IL‐4 (20 ng mL^−1^), IL‐13 (20 ng mL^−1^) (MedChemExpress, #HY‐P70460) for 48 h to obtain M2 TAMs. Afterward, immature DCs and M2 TAMs cells were respectively stimulated with LPS (20 ng mL^−1^) and LPS (100 ng mL^−1^) for 24 h. At the same time, the cells were intervened with or without the addition of rMIF (200 ng mL^−1^) (TargetMol, #TMPY‐02342). Splenocytes from C57BL/6 mice were isolated aseptically, mononuclear lymphocytes were isolated by peripheral blood lymphocyte isolation solution, and then CD8^+^ T cells were obtained by whole culture induction with KBM581 containing aCD3 (2 µg mL^−1^) (Biolegend, #100 339) and aCD28 (2 µg mL^−1^) (Biolegend, #102 115) antibody, and NK cells were obtained by whole culture incubation with KBM581 containing IL‐2 (20 ng mL^−1^) (PeproTech, #212‐12). Tregs were obtained by whole culture induction with KBM581 containing aCD3 (2 µg mL^−1^), aCD28 (2 µg mL^−1^) antibody, lL‐2 (20 ng mL^−1^), and IL‐4 (40 ng mL^−1^), all of which were cultured in 6‐well plates for 48 h respectively, with or without the addition of rMIF (200 ng mL^−1^) intervention.

### Animals

Female BALB/c mice aged 4–5 weeks and with a weight of ≈20 mg were purchased from Guangdong Medical Laboratory Animal Center. All animals were monitored for abnormal behaviors to minimize animal pain and suffering. All animal studies were performed by animal protocol procedures of Southern Medical University and approved by the Institutional Animal Care and Use Committee (IACUC), and the approval number is SMUL202405001.

### RNA‐Sequencing

RNA sequencing performs high‐throughput sequencing on the total RNA of different samples, screens differentially expressed genes and performs Gene Ontology (GO) enrichment analysis to obtain the biological process and pathway between different samples. An RNA sequencing was performed on tumor tissue at the end of treatment. Herein, NF‐1 was considered as the treatment group, compared with the control group (PBS and NF‐2). Each group had three independent replicates. RNA‐sequence analysis results were obtained from Shanghai Majorbio Bio‐pharm Technology Co., Ltd.

### Bioinformatics Analysis

The gene set “GOBP_GOLGI_ORGANIZATION” was downloaded and 157 GA‐related genes were obtained from the MSigDB database (https://www.gsea‐msigdb.org/gsea/msigdb/). Subsequently, the STRING database (https://cn.string‐db.org/) was utilized to construct a protein–protein interaction (PPI) network of these genes, which was subsequently visualized using Cytoscape. PubMed was searched, Web of Science, and Scopus from inception to September 2024 and collected original studies to identify molecular partners of P115 that have been discovered so far. MIF protein expression in BRCA and COAD was from the HPA database (https://www.proteinatlas.org/). Survival curves were analyzed in the PanCanSurvPlot online website database (https://smuonco.shinyapps.io/PanCanSurvPlot/). The spatial transcriptome analysis was obtained from the SpatialTME database (http://www.spatialtme.yelab.site/) and the SCAR database (http://scaratlas.com). Single‐cell sequencing analysis was conducted in the SCAR database. Gene mutation analysis was conducted by Sangerbox 3.0 (http://vip.sangerbox.com/). Immune infiltration analysis was performed using the TIMER database (http://timer.cistrome.org/). TIDE scores were performed from the TIDE database (http://tide.dfci.harvard.edu). A pan‐cancer analysis of MIF expression with comparisons between tumor and normal tissues was conducted in the TIMER database (https://cistrome.shinyapps.io/timer/).

### Statistical Analyses

The statistical graphs were performed by using Microsoft Excel 2016 software (Microsoft, Redmond, WA). The results are expressed as mean ± standard deviation (SD). The statistical difference between the experimental groups was analyzed by One‐way ANOVA, and when the *p* < 0.05, it was considered to have statistical significance.

Other experimental methods can be found in Supporting Information.

## Conflict of Interest

The authors declare no conflict of interest.

## Supporting information



Supporting Information

## Data Availability

The data that support the findings of this study are available from the corresponding author upon reasonable request.
