# Supplementary material for: Tailoring Tumor Cell Golgi Apparatus‐Targeting Self‐Assembled Peptide for Effective Immunotherapy via Reshaping MIF‐Mediated Immunosuppressive Network
Source: Adv Sci (Weinh). 2025 Feb 5;12(12):2415133. doi: 10.1002/advs.202415133 (PMC11948030; doi:10.1002/advs.202415133)
Supplement: Supplementary file 1 — Supporting Information [file ADVS-12-2415133-s001.docx]

**Supporting Information**

**Tailoring Tumor Cell Golgi Apparatus-Targeting Self-assembled Peptide for Effective Immunotherapy via Reshaping MIF-Mediated Immunosuppressive Network**

*Xiang Li^#^, Chengxinqiao Wang^#^, Junhan Liu^#^, Guifang Deng, Yongqiang Deng, Fang Hu, Yupeng Wang, Dongfang Zhou^*^*

Dr. C. Wang, X. Li, J. Liu, G. Deng, Prof. Y. Wang, Prof. D. Zhou

NMPA Key Laboratory for Research and Evaluation of Drug Metabolism & Guangdong Provincial Key Laboratory of New Drug Screening & Guangdong-Hongkong-Macao Joint Laboratory for New Drug Screening

School of Pharmaceutical Sciences

Southern Medical University

Guangzhou 510515, P. R. China

E-mail: dfzhou@smu.edu.cn

Prof. D. Zhou

Key Laboratory of Mental Health of the Ministry of Education

Southern Medical University

Guangzhou 510515, P. R. China

Dr. C. Wang, Prof. D. Zhou

Department of Ultrasonic Diagnosis & Orthopedic and Traumatology

Zhujiang Hospital

Southern Medical University

Guangzhou 510515, P. R. China

Prof. Y. Deng

Department of Pathophysiology, Guangdong Provincial Key Laboratory of Proteomics

School of Basic Medical Sciences

Southern Medical University

Guangzhou 510515, P. R. China

Prof. F. Hu

Biomaterials Research Center

School of Biomedical Engineering

Southern Medical University

Guangzhou 510515, P. R. China

^#^ These authors contributed equally to this work.

**Experimental details**

**S1. Materials**

2-Chlorotrityl Resin, Fmoc-Arg(pdf)-OH, Fmoc-Val-OH, Fmoc-Phe-OH, Fmoc-Lys (Boc)-OH, Fmoc-Lys (Dde)-OH, Fmoc-Leu-OH, Fmoc-Gly-OH, Fmoc-Asp (O*t*Bu)-OH, 1-Hydroxybenzotriazole (HOBT), O-Benzotriazole-N, N, N', N'-tetramethyluroniumhexaf (HBTU) were obtained at GL Biochem (Shanghai, China) Ltd. Tris was provided by AVT (Shanghai, China) pharmaceutical Tech Co., Ltd. 3-(4,5-dimethylthiazol-2-yl)-2,5-diphenyltetrazolium bromide (MTT) and 4’,6-diamidino-2-phenylindole dihydrochloride (DAPI) were purchased at Beijing Solarbio Science &Technology Co., Ltd. Dulbecco's modified eagle medium (DMEM), fetal bovine serum (FBS), penicillin-streptomycin solution and trypsin-EDTA solution penicillin, streptomycin and Triton X-100 were purchased from Life Technologies. KBM581 medium was purchased from Corning. All reagents used were analytical grade without further purification. Water was processed using a Master Touch-RUVF purification system (HHitech, Shanghai) with a minimum resistivity of 18.2 MΩ cm.

**S2. Experimental instrument**

The ^1^H NMR spectra were recorded on a Bruker AVANCE III 400 MHz spectrometer. Fluorescence spectra were obtained on an RF-6000 spectrometer (Shimadzu). Electron spray ionization mass spectrometry (ESI-MS) values were recorded on a 2010 LC-MS mass spectrometer (Shimadzu). High-performance liquid chromatography (HPLC) purification was performed on a HP PLUS 100D system equipped with double high-pressure pumps and a UV preparative detector using a Sepax GP-C18 column, with CH_3_OH (0.1% of TFA) and water (0.1% of TFA) as the eluent. Dynamic light scattering (DLS) experiments were determined by a Zetasizer Nano ZS instrument (Malvern, UK). Fourier transform infrared (FTIR) spectroscopies were obtained on a Nicolet IS50 FT-IR Spectrometer. The circular dichroism (CD) spectra were recorded on a Chirascan spectropolarimeter (Chirascan, Applied Photophysics). Transmission electron micrography (TEM) images were collected on a HT7700 Exalens microscope operated at an acceleration voltage of 120 kV.

Cell images were observed on a ZEISS Laser Scanning Microscope (LSM880) and N-SIM/N-STORM confocal laser scanning system. FCM analysis was conducted with BD LSRFortessa flow cytometer and FlowJo Software (TreeStar, Ashland, OR). The in vivo imaging of mice was performed with FX Pro (Bruker, America) in vivo imaging system. RNA-sequence analysis results were obtained from Shanghai Majorbio Bio-pharm Technology Co., Ltd. Hematoxylin and eosin staining (H&E), TdT-mediated dUTP Nick-End Labeling (TUNEL), and immunohistochemical staining were performed by huayin healthcare company (Guangzhou Huayin Healthcare Group Co., Ltd., Guangzhou, China).

**S3. Methods**

**Furin-responsiveness of peptide self-assemblies**

Prepared self-assemblied NF-1 (100 µM) was added to Tris-HCl buffer solution containing furin (0.5 µg/mL, pH 7.2 ~ 7.4, 25 mM Tris-HCl, 1 mM CaCl_2_, 1 mM glutathione) and then shaken at 37°C. The count rates were tested at different time points. The same experimental protocol was also applied to NF-2.

**Fluorescence spectroscopy of NF-1_RhB_**

The standard curve of NF-1_RhB_ is calculated by its fluorescence intensity. The NF-1_RhB_ solution with a series of concentrations was prepared by diluting the prepared solution with a ratio of ddH_2_O and methanol, which is 1. Then, the fluorescence emission of each solution was measured at room temperature, and emission was recorded from 560 to 750 nm (λex = 550 nm).

**Cell culture**

Mouse breast cancer 4T1 cells, Mouse colon cancer CT26 cells, and normal liver L929 cells were bought from the Institute of Biochemistry and Cell Biology, Chinese Academy of Sciences. 4T1 cells were maintained in Roswell Park Memorial Institute 1640 (RPMI 1640) supplemented with 10% (v/v) fetal bovine serum (FBS), 1% 100 U/mL penicillin, and 100 μg/mL streptomycin (all purchased from Hyclone Laboratories, Inc., Logan, UT, USA) at 37°C in a humidified 5% CO_2_ incubator. The medium of CT26 cells and L929 cells were routinely cultured in Dulbecco’s modified Eagle’s medium (DMEM), and other cultural conditions were the same as 4T1 cells.

**Cellular uptake**

To evaluate the cellular uptake, 4T1 cells, and L929 cells were seeded separately in a 24-well plate containing 14 mm cover glass (10^4^ cells per well), then incubated with NF-1_RhB_ (2 μM) for 8 h. Later, the cells were washed with PBS twice, fixed with 4% paraformaldehyde (PFA) for 20 min, and stained with DAPI (10 µg/mL) for 10 min. The fluorescence images of NF-1_RhB_ (λex = 550 nm) and DAPI (λex = 405 nm) were collected accordingly using CLSM. After incubation, these cells were washed with PBS three times before digestion using trypsin. The cell suspension was centrifuged at 3000 rpm, 5 min at 4°C, and re-suspended in PBS (0.3 mL). FCM analyzed the fluorescence intensity in cells. The excitation wavelength was 550 nm.

**Morphology of GA**

4T1 cells were seeded separately in a 6-well plate (2×10^6^ cells per well), then were incubated with NF-1 (100 μM) or NF-2 (100 μM) for 24 h. After washing with PBS three times, the cells were collected and formed into cell clumps. Followed by cell clumps, the cells were fixed with 2.5% glutaraldehyde for 1 h at room temperature and then fixed at 4°C for 3 h. Subsequently, cells were washed three times with PBS, dehydrated, macerated, embedded, and stained. Finally, the morphology of the GA was visualized by bio-TEM.

**RUSH assay**

The principle is that the target protein has a streptavidin binding domain, which is retained by endoplasmic reticulum streptavidin after expression and can then be released into the GA and secreted towards the plasma membrane after the addition of biotin. Therefore, rush-eqsm-gpf plasmid was constructed. 4T1 cancer cells were seeded into a 24-well plate containing 14 mm cover glass at the density of 10^4^ cells. After cells adhered, RUSH-eqsm-gpf plasmid transfected cells for 5 h. After incubation separately with culture medium and NF-1 (100 μM) for 24 h, the cells were added to a biotin solution for 60 min. Furthermore, the cells were washed three times with PBS, fixed with 4% PFA for 20 min, and stained with DiD dye (6 μM) for 20 min and DAPI (10 µg/mL) for 10 min. Finally, the cells were visualized by CLSM (LSM880) at 633 nm excitation for DiD, 488 nm excitation for plasmid, and 405 nm excitation for DAPI, respectively.

**Apoptosis assay**

The apoptosis induced by peptides was detected using an Annexin V-FITC/PI Apoptosis Detection Kit according to the manufacturer’s instructions. Briefly, after incubation separately with culture medium, NF-1 (100 μM) and NF-2 (100 μM) for 24 h, the cells (5 × 10^4^) were digested with 0.25% trypsin without ethylene diamine tetraacetic acid (EDTA), harvested with low-speed centrifugation, washed with PBS, and incubated with Annexin V-FITC (5 μL) and PI (10 μL) in binding buffer (400 μL) for 10 min in the dark at room temperature. The stained cells were analyzed using FCM.

**Fluorescence quantification inside GA**

Briefly, 4T1 cells were seeded in a 24-well plate and allowed for attachment at 37°C, 5% CO_2_. The cultural medium was removed, and a new medium containing 15 μM or 30 μM of NF-1_RhB_ was allowed to have sufficient cellular uptake by incubating at 37°C. After 3 h, the medium was removed, and cells were washed with cold PBS (three times). Using trypsin solution, 4T1 cells were harvested and collected in a falcon tube. The average cell number was determined using a cell counter. The cells were centrifuged at 1400 rpm for 5 min, collected as a pellet, and discarded as a supernatant. The pellet was gently suspended in PBS and centrifuged again, and the supernatant was discarded. The cells were collected as a pellet, and RIPA cell lysis buffer (200 μL) was added and maintained for 30 min; after ensuring the complete lysis of the cell, MeOH (200 μL) was added for the clarity of the spectra. The supernatant was collected by centrifugation and used for the experiment. The fluorescence emission spectra for these samples were measured (λex = 550 nm). A calibration curve was plotted initially for NF-1_RhB_ at different concentrations ranging from 0.03 μM to 1 μM in a 1:1 mixture of buffer and MeOH (200 μL of MeOH and 200 μL of RIPA lysis buffer) and plotted a calibration curve with the value of measured fluorescence intensity against concentration. Using this calibration plot, the concentration of NF-1_RhB_ in each of the collected supernatants was determined, which provides the amount of sample uptake by the cells. Then, the intracellular concentration was calculated by using the relationship: GA accumulation of NF-1_RhB_ = Cellular uptake (μ mol) / (Cell number × Volume of the GA of total 4T1 cells × co-localization coefficient of GA with NF-1_RhB_). The average size of the 4T1 cells is 20 μm, and the average cell volume for a single 4T1 cell was calculated as 4000 μm^3^. The volume ratio of the GA in every cell is about 0.0083.

**Cytotoxicity assay**

The cytotoxicity of NF-1 and NF-2 was examined using an MTT assay. 4T1 cells, CT26 cells and L929 cells were incubated in 96-well culture plates (5×10^3^ cells/well) for 12 h to adhere. Then, the cells were conducted using NF-1, NF-2, and NF-3 (0, 20, 40, 80, 100, 200, 400, 600 μM) for 24 h or 48 h, respectively. After 24 h or 48 h incubation, the cells were incubated with 10 μL MTT (5 mg/mL) solution at 37°C for another 4 h, and formazan crystals were dissolved in DMSO (150 μL). Finally, the absorbance was measured at 490 nm using a microplate reader (Tecan, Hombrechtikon, Switzerland). Cell viability was expressed as Cell viability (%) = (OD_sample_ − OD_blank_) / (O_Control_ − OD_blank_) × 100.

**Tumor inhibition experiments**

To develop tumors in BALB/c mice for BRCA, 1×10^7^ 4T1 cells were suspended in PBS (100 µL) and were subcutaneously implanted in the lateral thigh of mice. When those tumors reached ∼100 mm^3^, the mice were randomly divided into three groups (n = 5). Animals were treated with saline, NF-2 (15 mg/kg), or NF-1 (15 mg/kg) through injection on day 1, day 3, day 5, and day 7. To evaluate the therapeutic efficacy, the volume of tumor tissues was monitored by measuring the perpendicular diameter with the caliper, and the estimated volume was calculated based on the following equation: volume of tumor tissues = 1/2 × length ×width^2^. The image and weight of isolated tumor tissues were recorded at the end of treatment. To develop tumors in BALB/C mice for COAD, 1×10^7^ CT26 cells were suspended in PBS (100 µL) and were subcutaneously implanted in the lateral thigh of mice. When those tumors reached ∼100 mm^3^, the mice were randomly divided into four groups (n = 5). Animals were treated with saline, *α*PD-L1 (5 mg/kg), NF-1(15 mg/kg), and NF-1 (15 mg/kg) + *α*PD-L1 (5 mg/kg) through injection on day 1, day 4, day 7, and day 10. All mice bearing 4T1 tumors or CT26 tumors of different groups were sacrificed at the end of treatment, and major organs (heart, spleen, lung, kidney, and liver) and tumors were separated.

**Analysis of immune cells in tumor tissues**

At the end of treatment, tumor tissues, spleen, and lymph nodes were excised and transferred to a dish and cut into small pieces (less than 1 mm^3^). The fragments were suspended in 1 mL of digestion solution (type I collagenase (400 μg/mL) and type IV collagenase (100 μg/mL) in RPMI-1640 medium containing 10% FBS) and incubated at 37°C for 0.5 h with persistent agitation. Then, cells were collected by centrifugation at 1500 rpm for 5 min and then filtered by a 200-mesh sieve. Cells were digested with erythrocyte lysate for 2 min and collected. Finally, cells were stained with fluorescence-labeled antibodies and analyzed by FCM. The same protocol was performed for the CT26 tumor-bearing model.

**Bio-distribution**

When 4T1 tumors reached about 300 mm^3^, 4T1 tumor-bearing Balb/c mice were injected intratumorally by NF-1_RhB_ (15 mg/kg) and sacrificed at designated time points (0, 4 h, 12 h, and 24 h), and the tissues including heart, liver, spleen, lung, kidney, and tumor were excised and imaged to characterize NF-1 distribution.

**H&E staining or TUNEL assay**

Tumor tissues were harvested, weighed, photographed, and repeatedly washed with PBS. These tumor tissues were fixed using 4% PFA. The slices of tumor and organs were stained with TUNEL, as well as H&E, to evaluate the therapeutic effect and systemic toxicity and then imaged by optical microscopy. H&E and TUNEL were performed by Huayin Healthcare Company (Guangzhou Huayin Healthcare Group Co., Ltd., Guangzhou, China).

**Immunohistochemical staining**

These tumor tissues were fixed using 4% PFA. Rabbit polyclonal anti-P115 and anti-MIF antibody as the primary antibody was used for the staining. Immunohistochemical staining was performed by Huayin Healthcare Company (Guangzhou Huayin Healthcare Group Co., Ltd., Guangzhou, China).

**Cytokine detection**

Serum samples were isolated from mice after various treatments and diluted for analysis. According to vendors’ instructions, IFN-*γ* and TNF-*α* were analyzed with ELISA kits.

**Hematological tests** & hemolysis assay

Blood samples collected from the eyeballs and stored in ethylenediaminetetraacetic acid (EDTA) coated tubes were employed for the hematological assay. Potential cytotoxic were reflected by the levels of biomarkers, including alanine aminotransferase (ALT), aspartate aminotransferase (AST), urea nitrogen (UREA), and creatinine (CREA) were determined using an automated biochemical analyzer. For hemolysis assay, the fresh blood from healthy BALB/c mice was collected and pure erythrocytes were obtained from the whole blood by centrifugation. Erythrocytes were mixed with NF-1 (100 μM) or NF-2 (100 μM) and then incubated for 24 h at 37 °C. All samples were centrifugation to separate the supernatant which was subsequently analyzed using a microplate reader set at an absorbance wavelength of 540 nm.

**Supplemental figures**


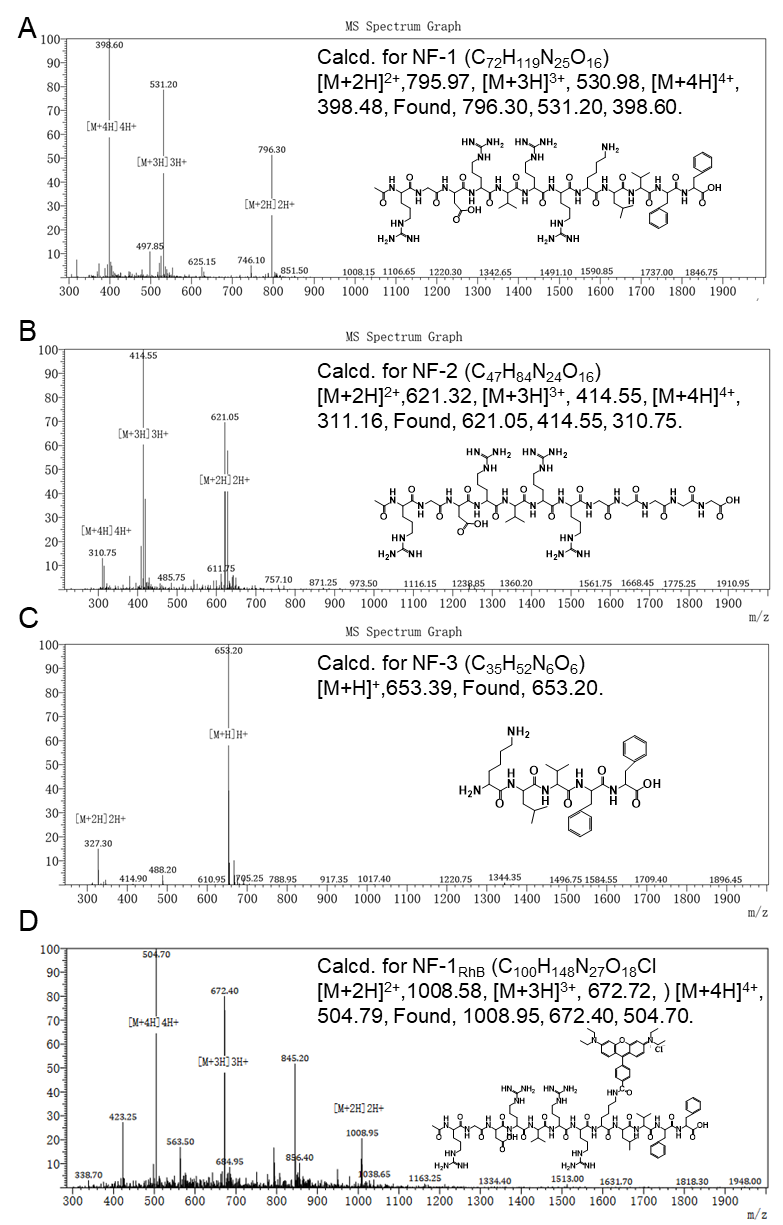


**Figure S1.** ESI-MS spectra of different peptides. A) NF-1. B) NF-2. C) NF-3. D) NF-1_RhB_.


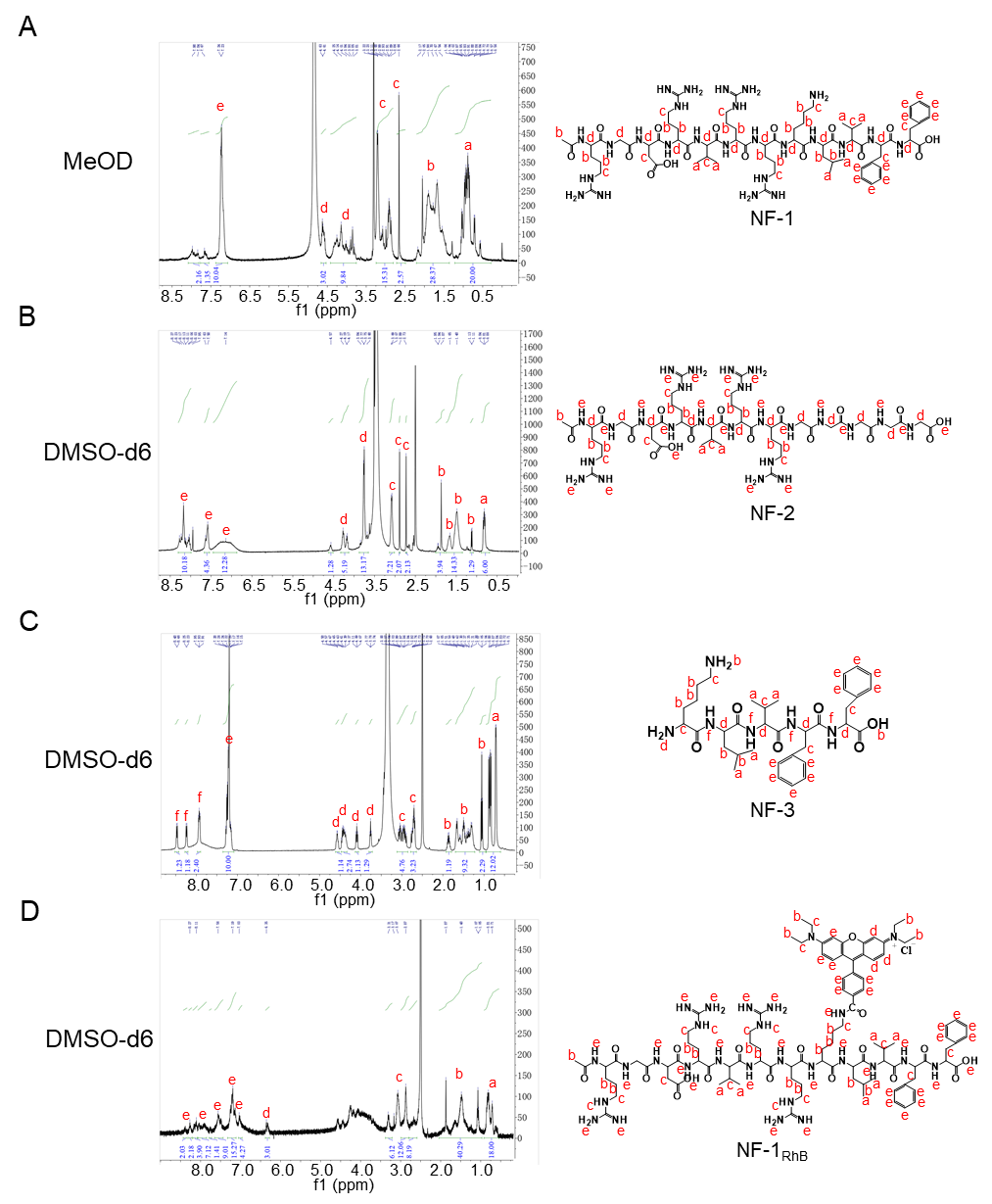


**Figure S2.** ^1^H NMR spectra of different peptides. A) NF-1. B) NF-2. C) NF-3. D) NF-1_RhB_.


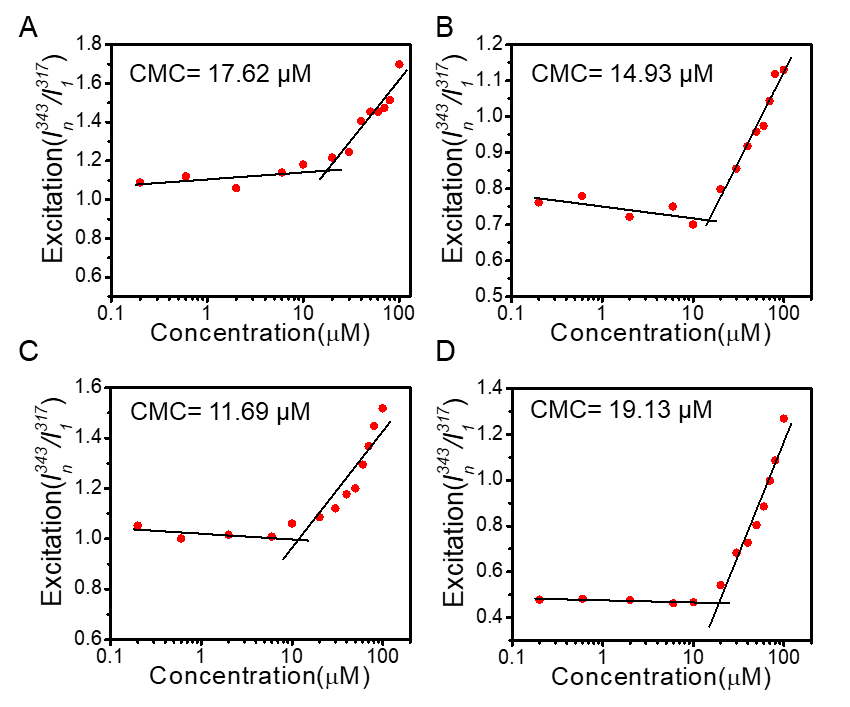


**Figure S3.** CAC characterization of different peptides in PBS solution. A) NF-1. B) NF-2. C) NF-3. D) NF-1_RhB_.

Figure S4. Hydrodynamic size and PDI changes of NF-1 incubated in PBS with 10% FBS at 37 °C for 7 days.


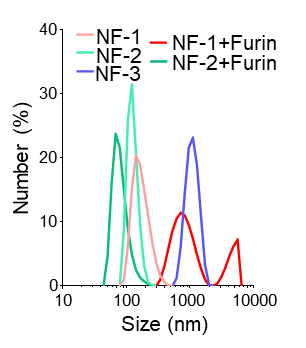


**Figure S5.** Hydrate diameters of NF-1, NF-2, and NF-3 self-assemble by DLS in the absence and presence of furin (0.5 µg/mL) for 12 h.


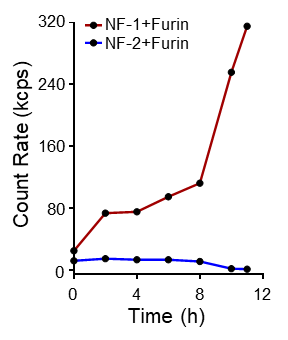


Figure S6. Count rates of NF-1 and NF-2 self-assemblies by DLS in the presence of furin (0.5 µg/mL) for 12 h.


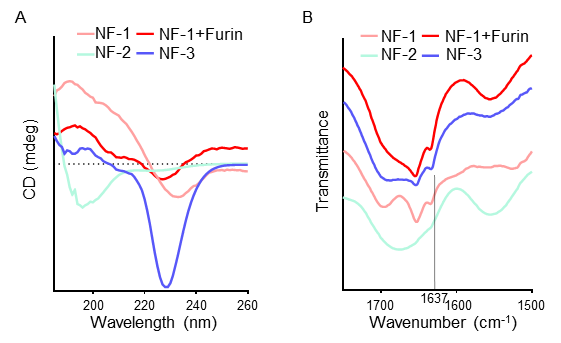


**Figure S7.** A) CD spectra and B) FTIR spectra of the self-assembled secondary structure of NF-1, NF-2, NF-3, and NF-1 treated with furin (0.5 µg/mL) for 12 h.


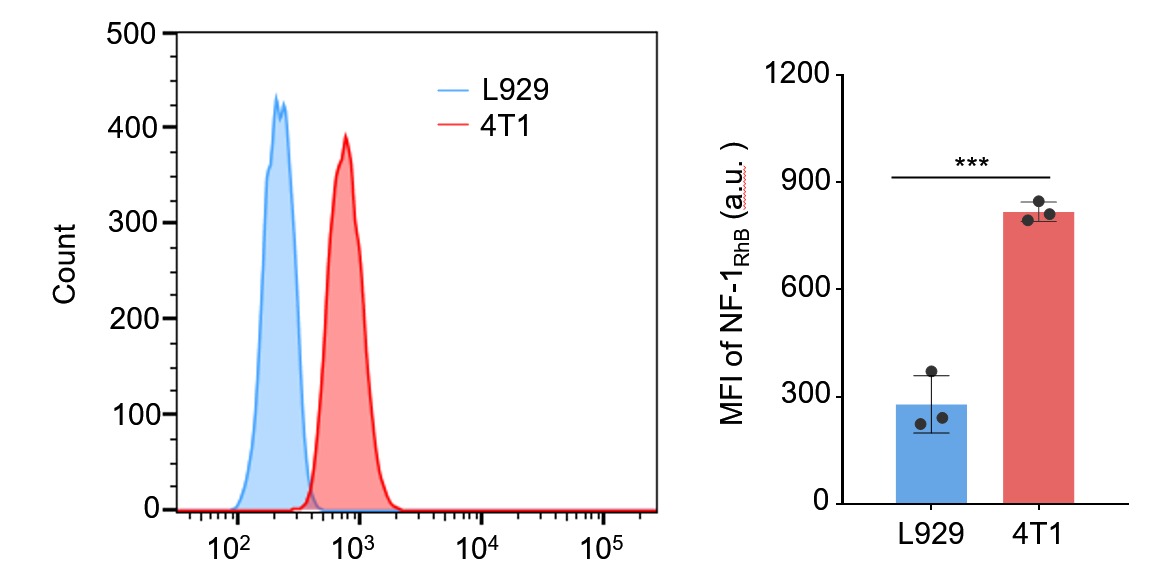


**Figure S8.** FCM analysis of L929 and 4T1 cells after coculture with NF-1_RhB_ (2 µM) for 8 h (n = 3 replicates).


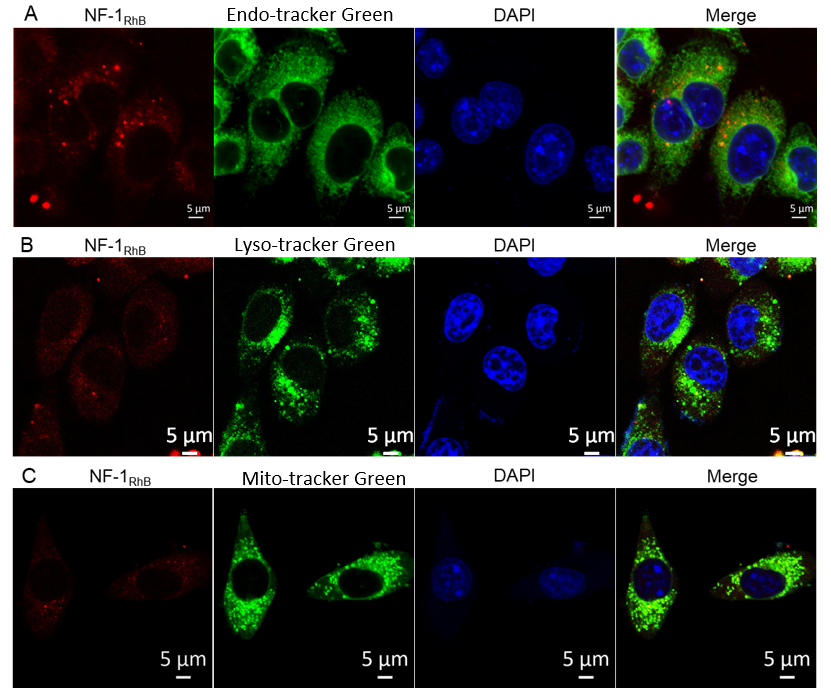


**Figure S9.** CLSM images of co-localization of NF-1_RhB_ (2 µM) and different subcellular organelle trackers in 4T1 cells after coculture for 8 h. A) Endoplasmic reticulum. B) Lysosome. C) Mitochondria. The scar bar is 5 µm.


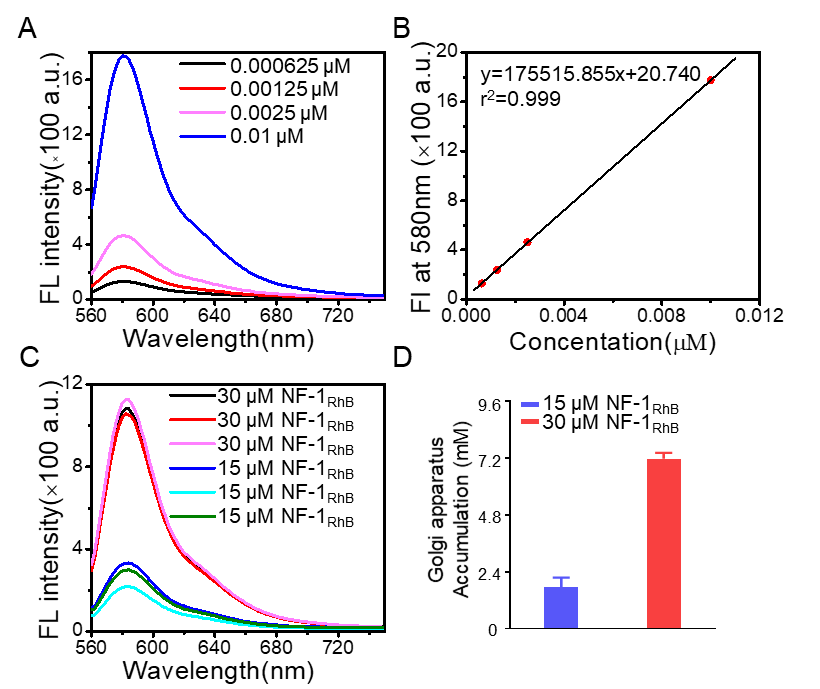


**Figure S10.** A) Fluorescence spectra of NF-1_RhB_ with different concentrations in buffer/MeOH mixture (1:1). B) Standard curve of NF-1_RhB_ according to fluorescent intensity at 580 nm. C) Fluorescent spectra of NF-1_RhB_ in the GA of 4T1 cells when the external incubation concentration is 15 µM or 30 µM. D) Quantitative accumulation of NF-1_RhB_ (15 µM or 30 µM) on GA in 4T1 cells after coculture for 3 h (n = 3 replicates).


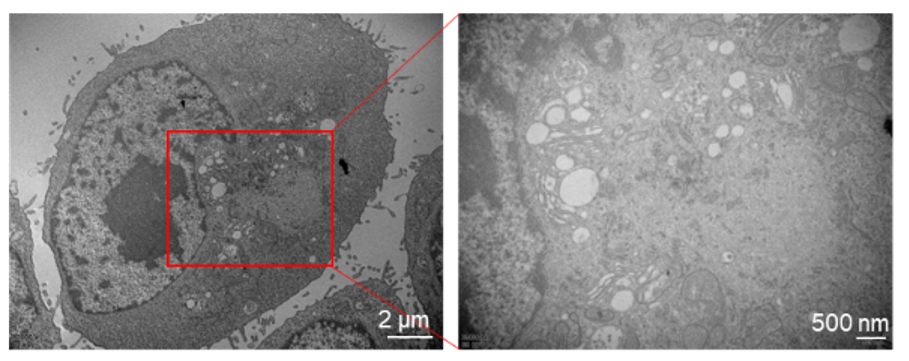


**Figure S11.** TEM images of 4T1 cells incubated with NF-1 (100 µM) for 2 h.


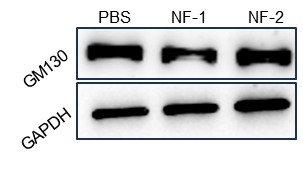


**Figure S12.** Total expression levels of GM130 proteins in 4T1 cells after different treatments by WB assay.


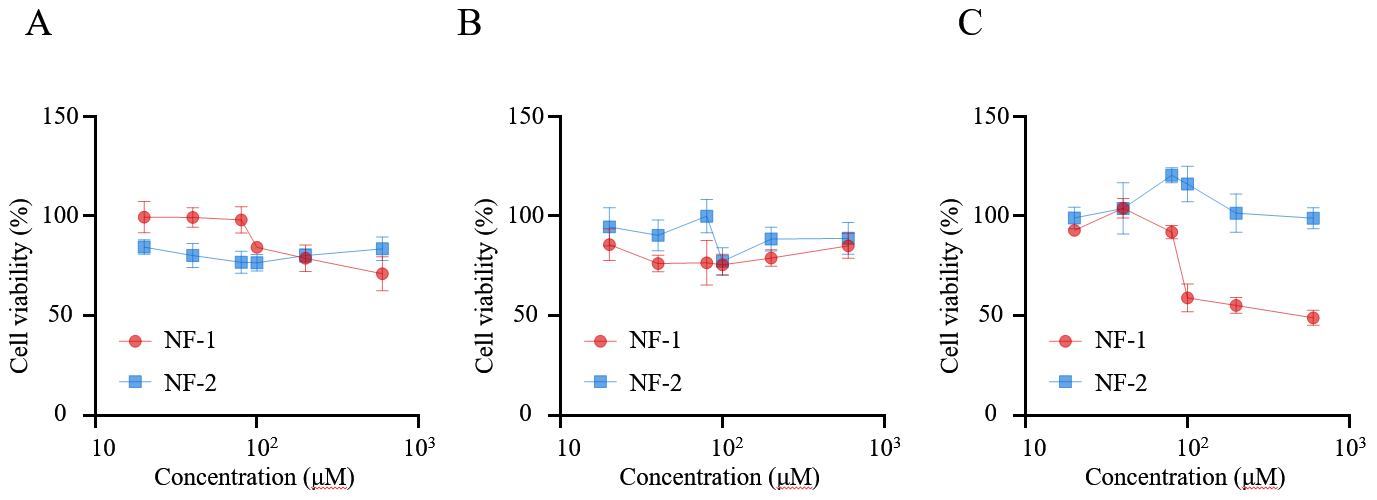


**Figure S13.** Cell viability curves of A) L929 cells (24 h) and B) L929 cells (48 h), and C) 4T1 cells (24 h) that were incubated with different peptides (NF-1, NF-2) at various concentrations (0, 20, 40, 80, 100, 200, 400, and 600 µM).


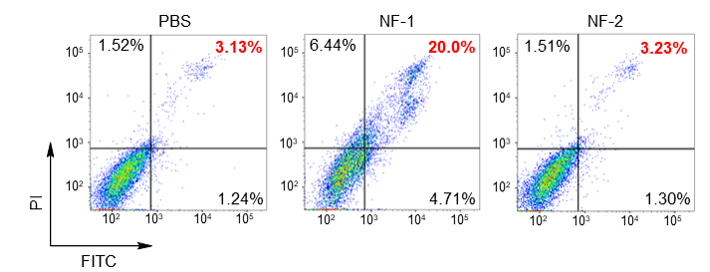


**Figure S14.** Apoptosis assay of 4T1 cells by FCM after incubation of NF-1 (100 µM), NF-2 (100 µM) and NF-3 (100 µM) for 24 h. Cells were stained by Annexin-FITC and PI.

**
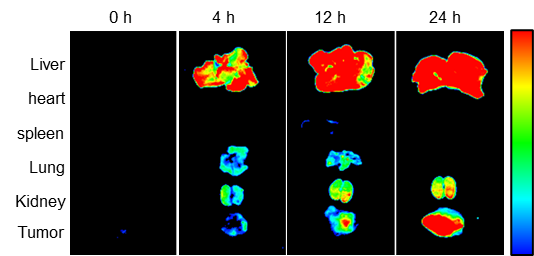
**

**Figure S15.** Bio-distribution of NF-1_RhB_ (20 mg/kg) after intravenous injection at different time points (0, 4 h, 12 h and 24 h) in 4T1 tumor-bearing model.


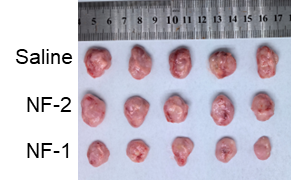


Figure S16. Tumor photographs of isolated tumor tissues of mice after treatments of saline, NF-2, and NF-1 (n = 5 replicates).


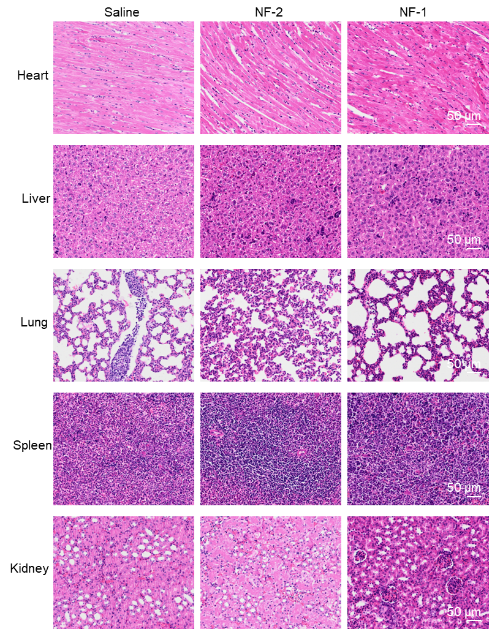


**Figure S17.** H&E staining images of the mice’s major organs (heart, liver, spleen, lung, and kidney) at the end of treatment. The scar bar is 50 µm.


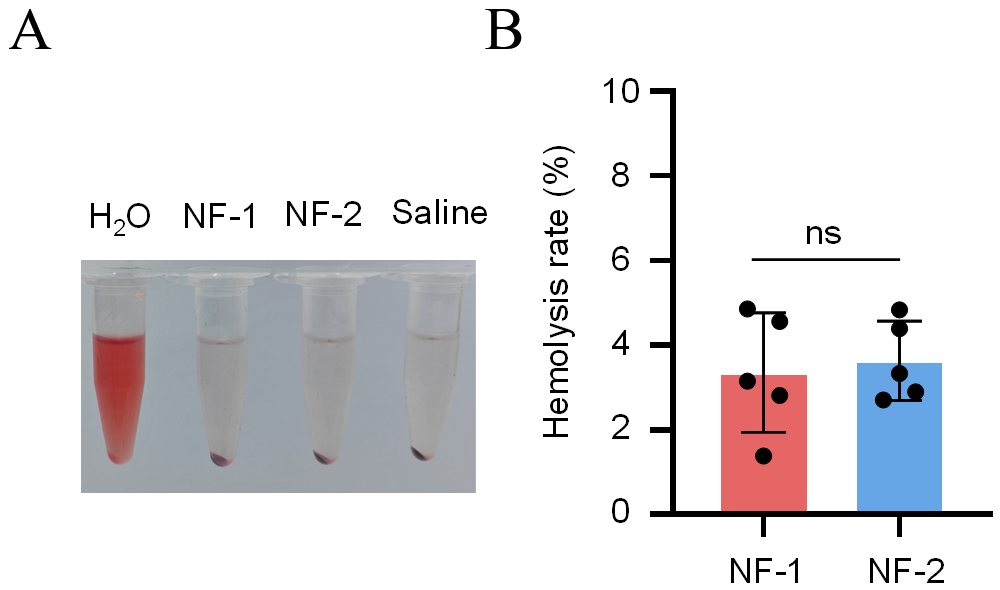


Figure S18. A) Representative image and B) statistical results of hemolysis assay.


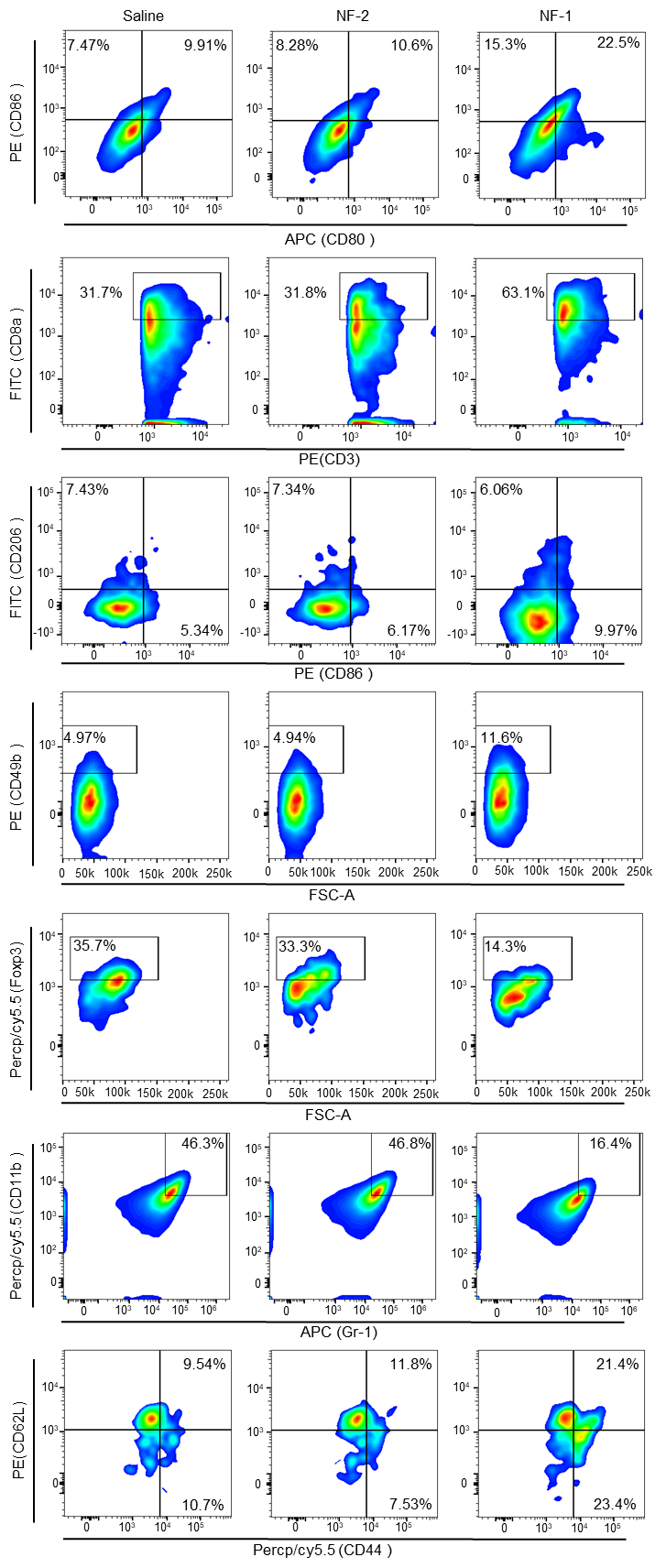


**Figure S19.** Representative FCM analysis of mDCs, CD8^+^ T cells, M1/M2, NK cells, Tregs, MDSCs, and Memory T cells after saline, NF-1, and NF-2 treatment in 4T1 tumor-bearing model.


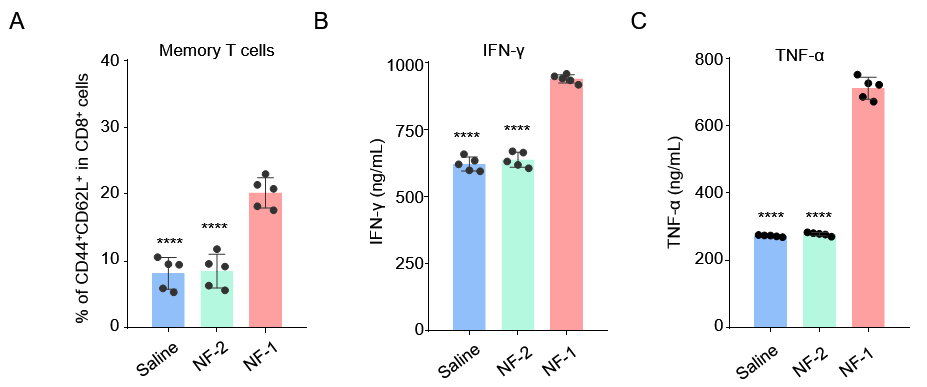


**Figure S20.** A) Statistical results of FCM analysis of Memory T cells after saline, NF-1, and NF-2 treatment. ELISA analysis of B) IFN-γ and C) TNF-α secretion after different treatments in 4T1 tumor-bearing model.


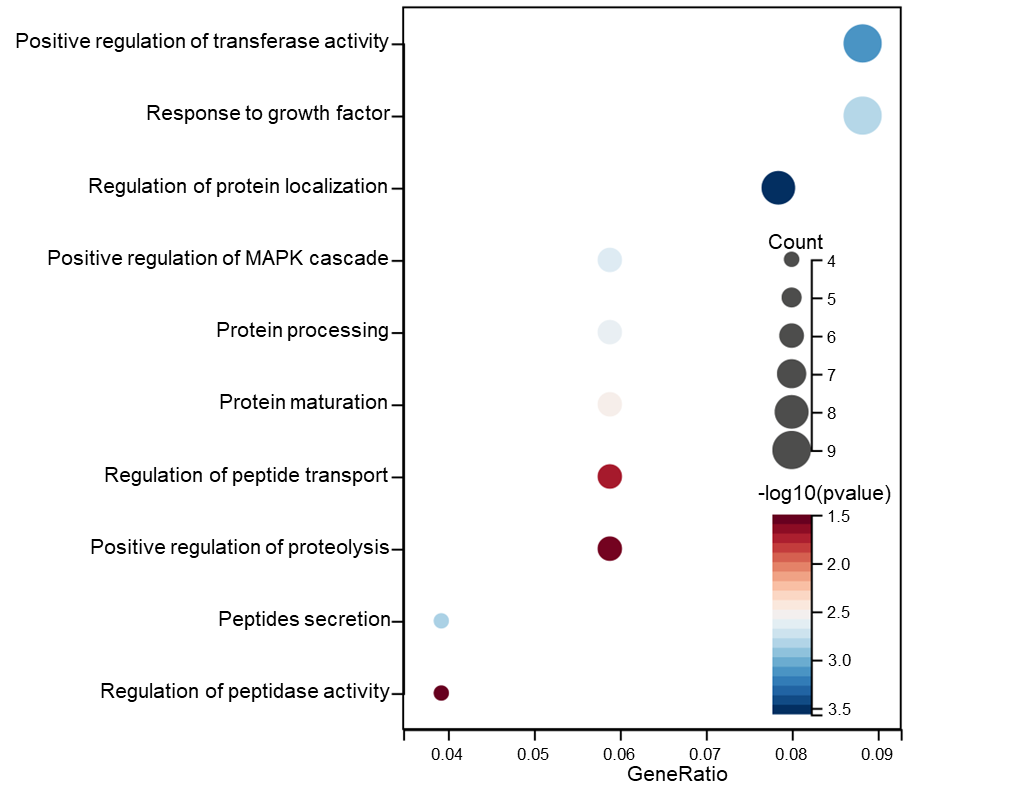


**Figure S21.** KEGG analysis of up-regulated genes.


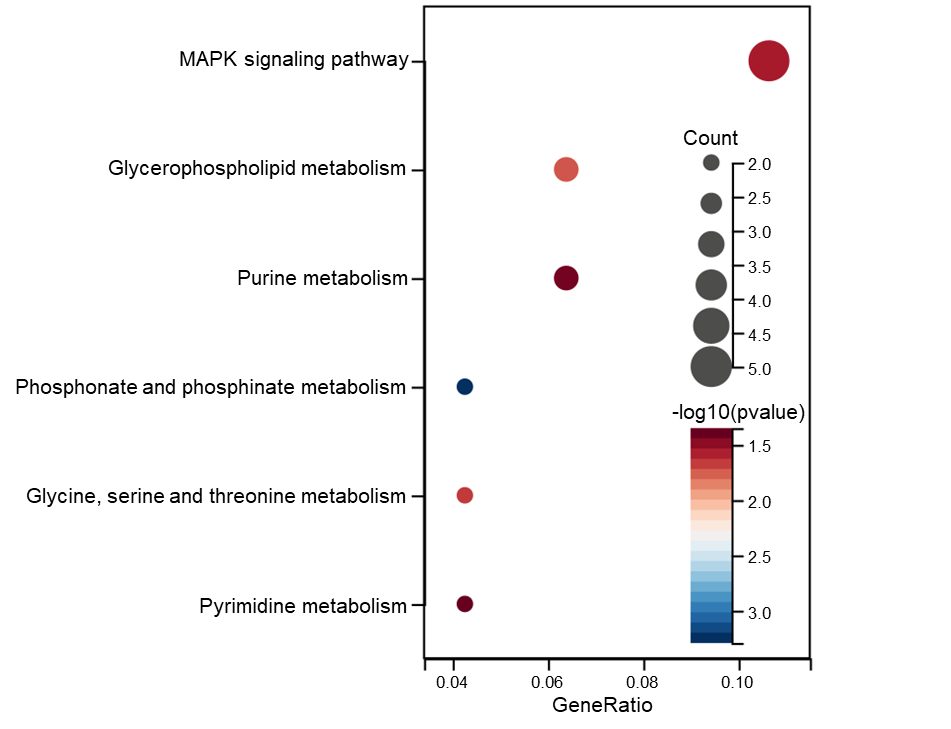


**Figure S22.** KEGG analysis of down-regulated genes.

**Figure S23.** Immunohistochemical staining of A) P115 and B) MIF in breast cancer tissues and

normal para-cancerous tissues of clinical specimens and corresponding statistical

graphs. Statistical P-values: * P < 0.05.

**Figure S24.** P115 protein expression in BRCA and normal breast tissue form HPA database.

**Figure S25.** **Multi-omics analysis reveals that tumor cell-derived GA-dependent MIF is closely related to TIME.** A) Spatial representation of cell types of TNBC from GSE176068 based on SpatialTME database. B) MIF spatial expression from GSE176068. C) The MIF expression in immune cells, malignant cells, and stromal cells of single cell datasets based on TISCH2 database. D) Spatial expression pattern of MIF, CD74, CXCR4, and CD44 in BRCA from SCAR_ST_000077 based on SCAR database. E) Cell distribution and MIF expression of TNBC from GSE148673 based on SCAR database. F) MIF-mediated cell interaction from GSE148673. G) The waterfall plot displayed the mutation information of top 5 mutated genes between MIF high- and low-expression groups. The annotation of mutation types was shown on the right with various colors.


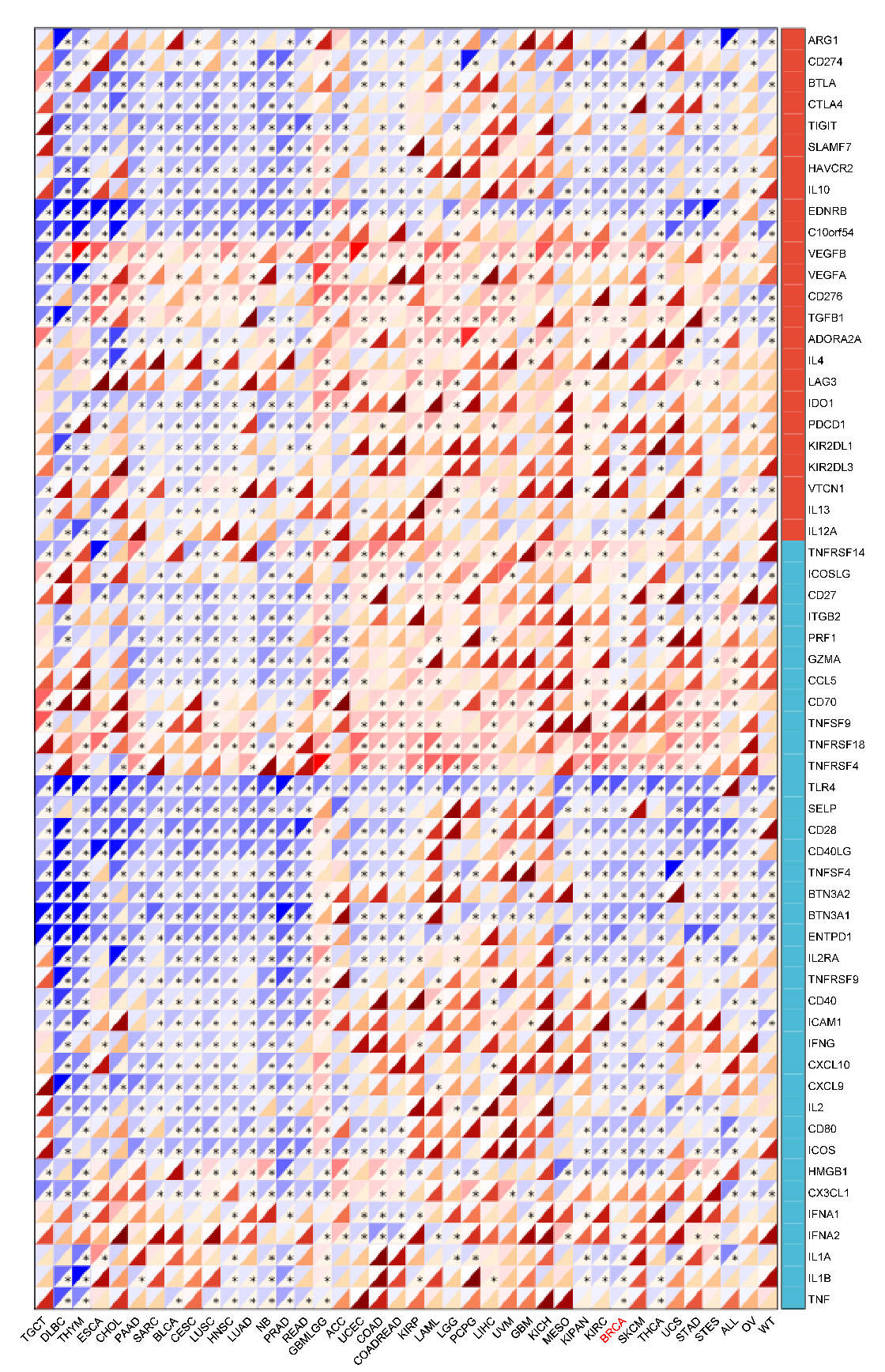

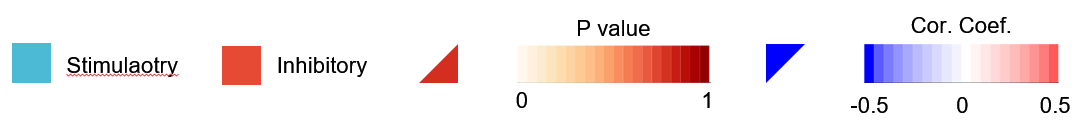


Figure S26. Correlation heatmap between MIF expression and immune checkpoint expression in pan-cancer.


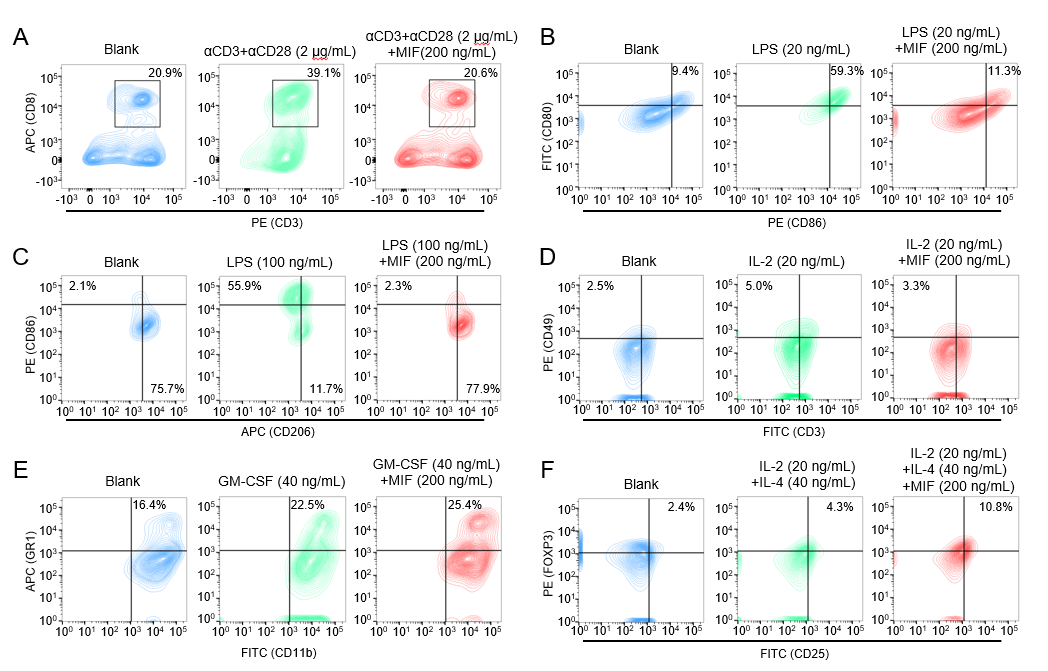


Figure S27. FCM analysis of different immune cell differentiation outcomes induced by different cytokines and recombinant MIF intervention. A) CD8^+^ T cells. B) mDCs. C) Macrophages. D) NK cells. E) MDSCs. F) Tregs.


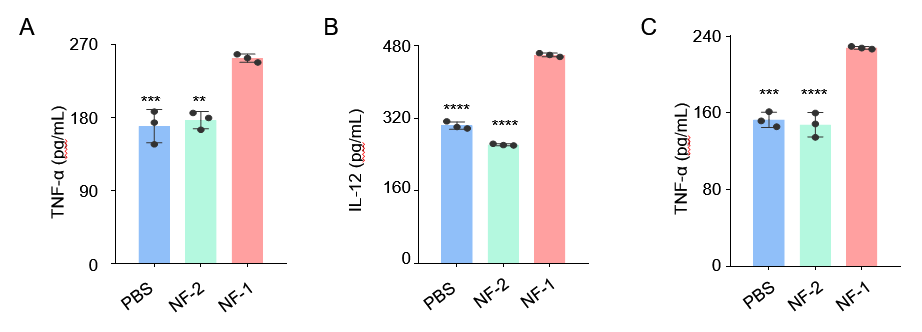


**Figure S28.** A) ELISA analysis of TNF-α secretion in CD8^+^ T cells after different treatments. B) ELISA analysis of IL-12 secretion in BMDCs after different treatments. C) ELISA analysis of TNF-α secretion in M1 cells after different treatments.


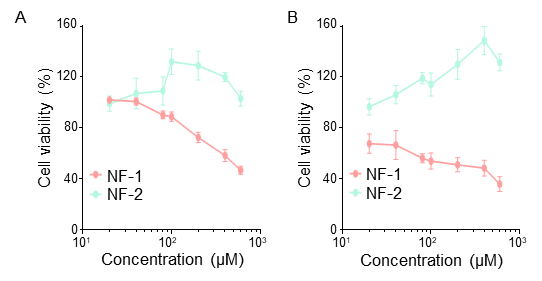


**Figure S29.** Cell viability curves of CT26 cells that were incubated with different peptides (NF-1, NF-2) at various concentrations (0, 20, 40, 80, 100, 200, 400, and 600 µM) for different times. A) 24 h. B) 48 h.


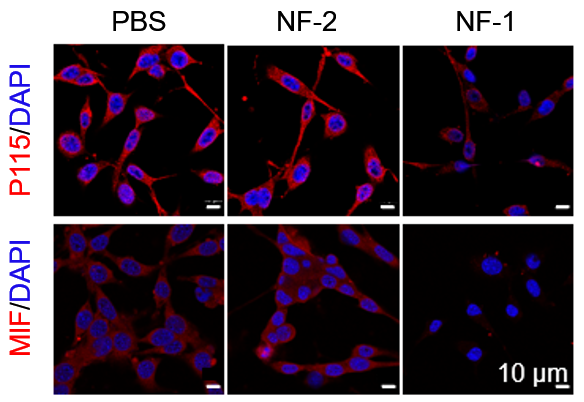


**Figure S30.** Immunofluorescence of P115 and MIF of CT26 cells after different treatments.


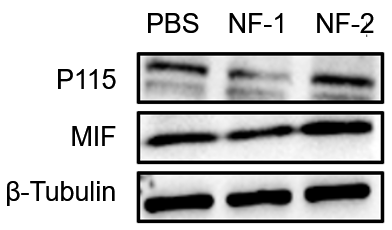


**Figure S31.** WB of P115 and MIF of CT26 cells after different treatments.


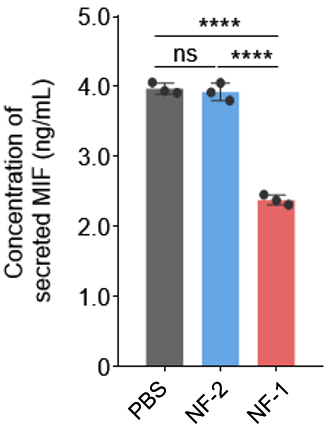


**Figure S32.** ELISA analysis of secreted MIF of CT26 cells in the supernatant after different treatments.


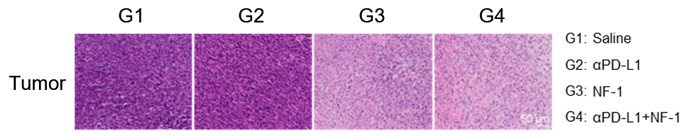


**Figure S33.** H&E staining image of CT26 tumor tissues at the end of different treatments. The scar bar is 50 µm.


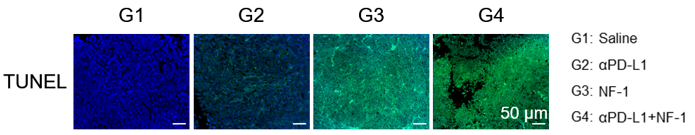


**Figure S34.** TUNEL staining of isolated CT26 tumor tissues at the end of different treatments. The scar bar is 50 µm.


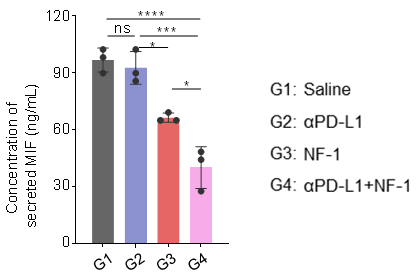


**Figure S35.** ELISA analysis of secreted MIF in CT26 tumor after different treatments.


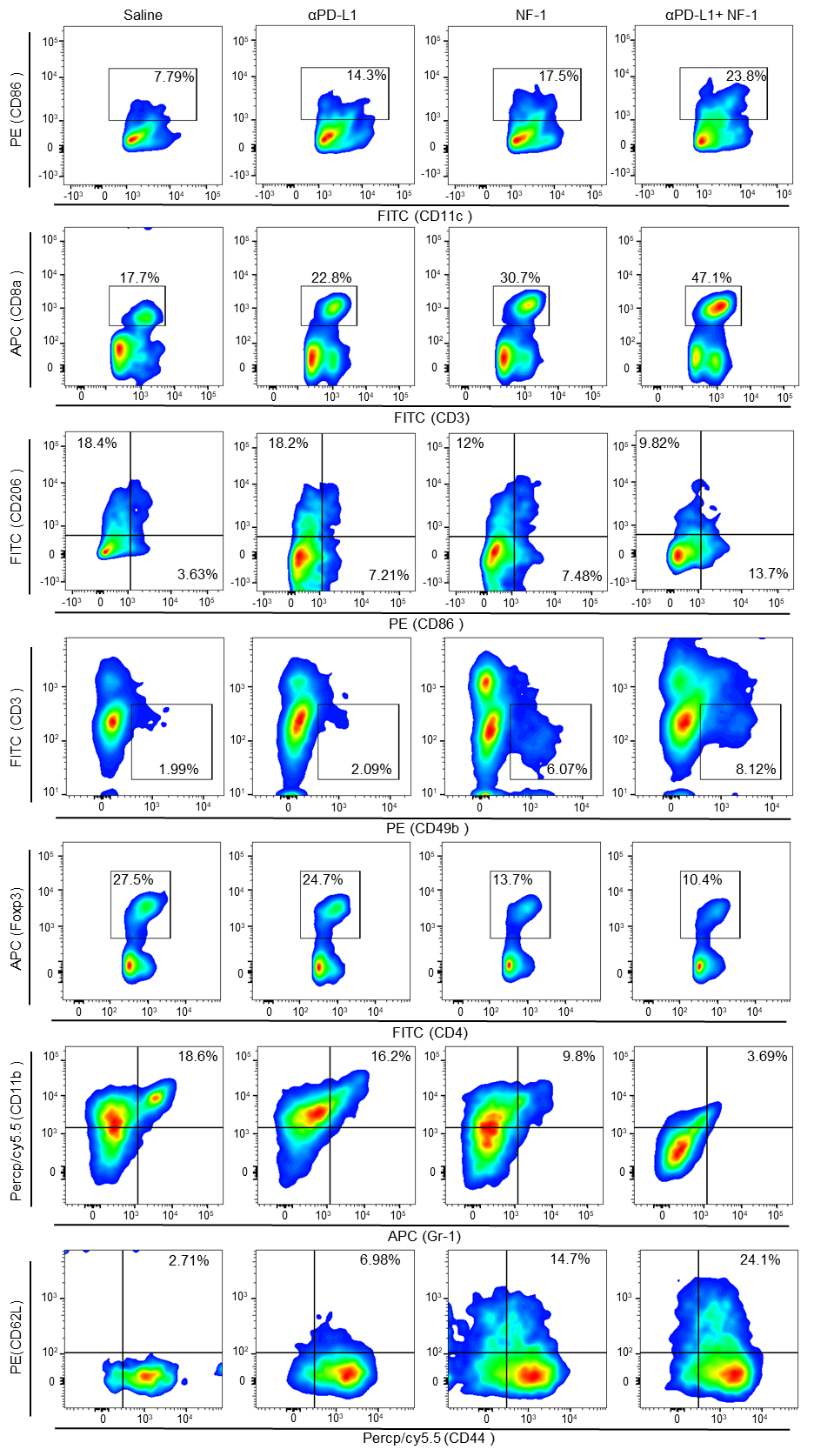


**Figure S36.** Representative FCM analysis of mDCs, CD8^+^ T cells, M1/M2, NK cells, Tregs, MDSCs, and Memory T cells after saline, *α*PD-L1, NF-1, and *α*PD-L1 + NF-1 treatment.


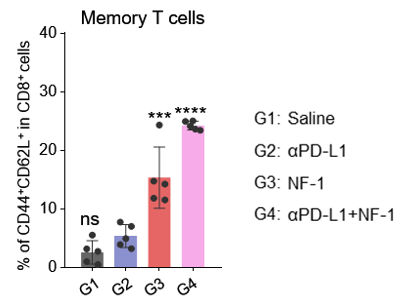


**Figure S37.** Statistical results of FCM analysis of Memory T cells after different treatments.

**
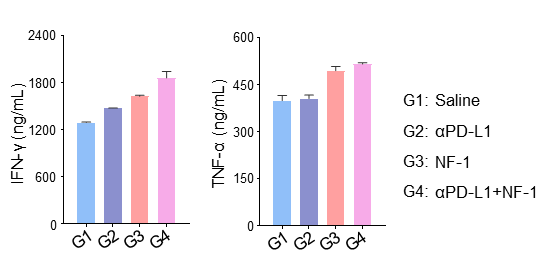
**

Figure S38. ELISA analysis of IFN-γ and TNF-α levels in serum at the end of CT26-tumor model treatments (n = 5 replicates).


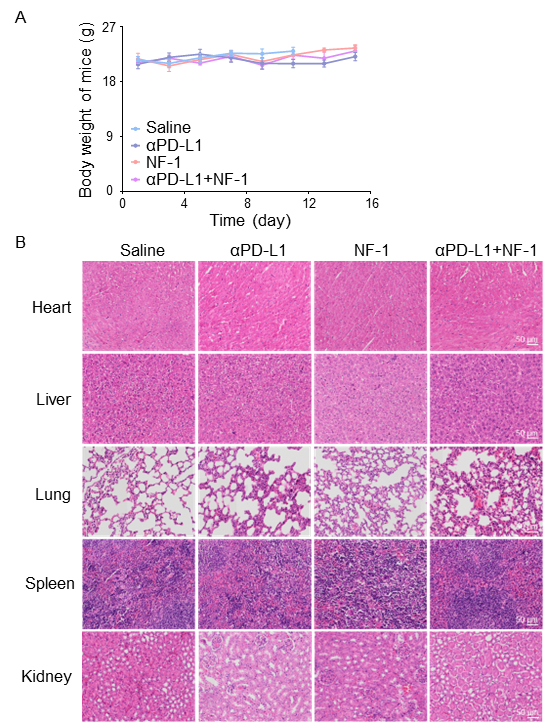


Figure S39. A) Body-weight curves of CT26 tumor-bearing mice. Data are expressed as means ± SD (n = 5). B) H&E staining images of the major organs (heart, liver, spleen, lung, and kidney) of CT26 tumor-bearing mice at the end of treatment. The scar bar is 50 µm.


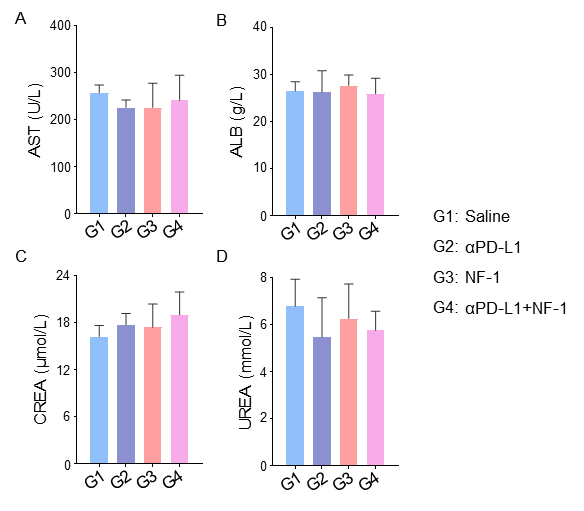


Figure S40. Serum biochemical analysis of CT26 tumor-bearing mice at the end of treatment. A) AST (aspartate aminotransferase). B) ALB (serum albumin). C) CREA (creatinine). D) UREA. Data are expressed as means ± SD (n = 5).

**Table S1**. 157 GA-Related Genes.

| GOLGA8DP | ARFGEF1 | PLK3 | VPS13B | CLASP1 | GOLGA2 | YWHAZ | RAB29 |
| --- | --- | --- | --- | --- | --- | --- | --- |
| HUWE1 | TMED2 | TBC1D20 | UBXN2A | SYNE1 | GOLGB1 | CORO7 | SEC16B |
| GOLGA8R | TMED10 | TANGO2 | BHLHA15 | OBSL1 | GOLGA8IP | ARMH3 | ZW10 |
| OPTN | TMED1 | UBXN2B | TMED4 | TMED3 | YIPF7 | DYNC2H1 | COG7 |
| AKAP9 | CRYZL2P-SEC16B | VTI1A | COG2 | GAK | HTT | ATP8B4 | TRIP11 |
| BET1 | SEC23IP | CSNK1A1 | TRAPPC8 | COG4 | EHD3 | VCPIP1 | TJAP1 |
| PRMT5 | PDCD10 | CSNK1D | GOLGA8A | ATL3 | RAB43 | YIPF5 | COG1 |
| COG5 | ZNF501 | TMED6 | CLASP2 | GORASP2 | GOLGA6A | VMP1 | BAG5 |
| VTI1B | LYSMD3 | ATP8B3 | PLEKHM2 | FBXW8 | KIFC3 | RAB1B | SEC22B |
| STK25 | LRRK2 | GARIN4 | CAMSAP2 | RAB30 | MYO18A | RAB33B | GCC2 |
| LMAN1 | SPTBN5 | GOLPH3L | MAP2K2 | RBSN | GOLGA6D | COG3 | PDE4DIP |
| ARL1 | HOOK1 | PI4K2B | ATP8B2 | ATL2 | GOLGA8M | YIPF4 | USP6NL |
| FAM174B | HIKESHI | PI4K2A | COG6 | GOLGA8N | STX5 | COG8 | CUL7 |
| RAB8A | RAB6B | NPLOC4 | HACE1 | GORASP1 | SURF4 | RAB2B | CDK1 |
| GOLGA8B | ATP8B1 | PRKD1 | ARHGAP21 | ZFP69B | GOLGA8Q | FHDC1 | SEC16A |
| MYO5A | STX18 | GOLGA6B | CAMSAP3 | GOLGA8S | GOLGA8O | BLZF1 | GOLGA5 |
| NAGLU | PLK1 | MAPK1 | RAB1A | GOLGA8J | GOLGA8H | USO1 | CDC42 |
| TMED5 | TMED9 | MAPK3 | RAB2A | GOLGA8T | GOLGA8CP | VAMP4 |  |
| TMED7 | DYM | NSFL1C | TRAPPC11 | GOLGA8K | VPS51 | GBF1 |  |
| TRAPPC12 | STX17 | MAP2K1 | GOLPH3 | GOLGA6C | VRK1 | ARHGEF7 |  |
